# An *in vitro* fluorescence based study of initiation of RNA synthesis by influenza B polymerase

**DOI:** 10.1093/nar/gkx043

**Published:** 2017-01-25

**Authors:** Stefan Reich, Delphine Guilligay, Stephen Cusack

**Affiliations:** 1 Grenoble Outstation, European Molecular Biology Laboratory, Grenoble 38042, France; 2 Unit of Virus-Host Cell Interactions, EMBL-UGA-CNRS, Grenoble 38042, France

## Abstract

Influenza polymerase replicates, via a complementary RNA intermediate (cRNA), and transcribes the eight viral RNA (vRNA) genome segments. To initiate RNA synthesis it is bound to the conserved 5΄ and 3΄ extremities of the vRNA or cRNA (the ‘promoter’). 5΄-3΄ base-pairing in the distal promoter region is essential to position the template RNA at the polymerase active site, as shown by a new crystal structure with the 3΄ end threading through the template entry tunnel. We develop fluorescence polarization assays to quantify initiation of cap-primed (transcription) or unprimed (replication) RNA synthesis by recombinant influenza B polymerase bound to the vRNA or cRNA promoter. The rate-limiting step is formation of a primed initiation complex with minimally ApG required to stabilize the 3΄ end of the template within the active-site. Polymerase bound to the vRNA promoter initiates RNA synthesis terminally, while the cRNA promoter directs internal initiation at a significantly lower rate. Progression to elongation requires breaking the promoter 5΄-3΄ base-pairing region and favourable compensation by the emerging template-product base-pairs. The RNA synthesis assay is adaptable to high-throughput screening for polymerase inhibitors. In a pilot study, we find that initiation at the cRNA promoter is unusually susceptible to inhibition by 2΄F-2΄dNTPs.

## INTRODUCTION

Influenza virus is a global public health threat with seasonal epidemics causing millions of cases of moderate to severe illness and 250–500 000 deaths annually (WHO, Fact sheet N°211, March 2014). Furthermore, especially virulent strains of influenza virus originate sporadically and unpredictably, either by spontaneous mutations or by genome segment re-assortment between existing viruses of human and/or animal origin (1–3). Influenza viruses are currently classified into four types, A, B, C and D (http://www.cdc.gov/flu/about/viruses/types.htm). Whilst influenza A is more variable and potentially more dangerous to humans due to its pandemic potential, influenza B is also clinically important and recently some vaccines have been augmented to contain two A strains and two B strains to take into account the divergence in the influenza B lineage (4). Current specific drug treatment of influenza virus infection focuses on inhibiting the receptor-destroying activity of the viral surface glycoprotein neuraminidase (5), which is required by the virus to cleave sialic acids from the infected cell surface to allow release of progeny virions. However, some circulating viruses have already acquired resistance, emphasizing the need for additional and differently targeted drugs to combat the virus efficiently (6). One promising target is the heterotrimeric viral RNA-dependent RNA polymerase (with subunits PA, PB1 and PB2), due to its central role in viral replication which occurs in the infected cell nucleus. The viral genome comprises eight segments of negative-sense single-stranded RNA, each coding for one, sometimes two, viral proteins. Each genome segment is individually packaged into a ribonucleoprotein particle (RNP), bound and protected by many copies of the viral nucleoprotein (NP) together with one copy of the polymerase. The polymerase binds to the highly conserved, near complementary, 5΄ and 3΄ ends of the genomic RNA (thus pseudo-circularizing it) and this constitutes the promoter where transcription and replication initiates. Recent high resolution crystal-structure of the promoter bound polymerase pre-initiation complex (7,8) show that nts 1–10 of the conserved 5΄ end forms an intramolecular stem–loop (‘hook’) which is anchored to the polymerase by binding to a special pocket formed between the PA and PB1 subunits. The distal parts of the 3΄ and 5΄ ends are base-paired, while the proximal 3΄ end needs to enter the active-site cavity to serve as the template and site for initiation of RNA synthesis. The polymerase performs transcription by a unique process known as cap-snatching (9,10). In the infected cell nucleus, the cap-binding domain within the PB2 subunit (11) binds to nascent capped Pol II transcripts which are then cleaved 10–15 nucleotides downstream by the endonuclease in the PA subunit (12–14) to generate a short capped RNA primer which can then be elongated by the polymerase. Viral mRNAs so transcribed are also poly-adenylated by the viral polymerase before nuclear export and translation. For replication, the polymerase initiates RNA synthesis on the same promoter but is unprimed (*de novo*) producing a full-length complementary, positive-sense anti-genomic RNA (cRNA). The cRNA co-transcriptionally assembles with polymerase and NP to form a cRNP (15) which is capable of unprimed synthesis of vRNA thus amplifying the genome. Finally, progeny vRNPs translocate to the plasma membrane to be incorporated into newly budding virions. Hence, during the viral ‘life’ cycle, the polymerase not only initiates RNA synthesis at the same vRNA-promoter either by using a primer or unprimed for transcription and replication, but furthermore initiates at the vRNA and cRNA promoter to replicate cRNA and vRNA, respectively. In contrast to terminal initiation of RNA synthesis at the vRNA promoter's 3΄ end, RNA synthesis at the cRNA promoter has been demonstrated to initiate internally at U4/C5 followed by a re-alignment of the short primer-product to the terminal 3΄ end prior to elongation (16).

Here, we establish a fluorescence polarization based *in vitro* RNA synthesis assay to quantitatively characterize primed and unprimed RNA synthesis by influenza polymerase using a model system comprising full-length recombinant influenza B polymerase, a promoter consisting of the separated conserved 5΄ and 3΄ (anti-) genome ends and no NP. Using the assay, we determined the enzymatic parameters of influenza B virus polymerase. We also address the mechanistic question of how the same polymerase initiates RNA synthesis either terminally or internally, directed only by the respective cRNA or vRNA promoter bound. By analyzing RNA synthesis from chimeric promoter RNAs, a cooperation of the 5΄-3΄ base-pairing region and the template-directed initial base-pairs was found to be responsible for allowing or preventing terminal initiation to proceed to elongation while an appropriate primer by-passed the rate-limiting formation of the first phosphodiester bond that yields ApG. This work is complemented by a new X-ray structure of influenza B polymerase co-crystallized with the vRNA promoter and a capped primer where we see for the first time 3΄ end of the template within the active site cavity. The assay we developed is easily adaptable to high-throughput-screening of compound libraries for polymerase inhibitors. By characterizing the inhibition profile of a set of nucleotide-analogues, we demonstrate the unusual susceptibility of polymerase initiating RNA synthesis from the cRNA promoter to 2΄F-2΄dNTPs and provide evidence that early initiation is more prone to inhibition than elongation.

## MATERIALS AND METHODS

### Protein preparation

The influenza B/Memphis/13/03 polymerase heterotrimer was expressed as a self-cleaving polyprotein and purified as described (8) with minor modifications. Briefly, High Five insect cells expressing the target protein were resuspended in buffer A (50 mM Tris/Cl, 500 mM NaCl, 10% (v/v) glycerol, 5 mM BME, pH 8) supplemented with protease inhibitors (Roche, complete mini, EDTA-free), lysed by sonication and centrifuged at 30 000 rpm for 30 min at 4°C (rotor type 45 Ti, Beckman Coulter). Ammonium sulfate was added to the clarified supernatant (0.5 g/ml), the resulting precipitate collected by centrifugation as above and re-dissolved in buffer A supplemented with 20 mM imidazol. Soluble proteins were loaded on a nickel nitrilo-triacetic acid column (GE, FF crude) and bound proteins were eluted by 500 mM imidazole in buffer A. The target protein was loaded on a streptactin matrix (IBA, Superflow) and bound proteins eluted by 2.5 mM d-desthiobiotin in buffer A. Fractions containing the target protein were pooled and diluted with two volumes of buffer B (50 mM HEPES/NaOH, 5% (v/v) glycerol, 2 mM Tris(2-carboxyethyl)phosphine (TCEP), pH 7.4) prior to loading on a heparin column (GE, FF). Proteins were eluted by a gradient of buffer B supplemented with 1 M NaCl. Homogeneous monomeric influenza B polymerase was concentrated (Amicon Ultra, 50 kDa MWCO) and stored in aliquots at −80°C. Protein concentration was determined by measuring the absorbance at 280 nm and using the extinction coefficient 287 300 M^−1^ cm^−1^.

### RNAs

Capped-RNAs with the sequence 5΄-m^7^GpppAAUCUAUAAUAG-3΄ (for enzymatic studies) or 5΄-m^7^GpppAAUCUAUAAUAGC-3΄ (for crystallographic studies) and the 2΄F-2΄dNTPs were purchased from TriLink BioTechnologies (San Diego, USA). All other RNAs used were purchased from IBA (Goettingen, Germany) with sequences listed in Supplementary Table S1. NTPs were purchased from Sigma.

### Crystallization and structure determination

For crystallization in hanging drops at 4°C, 35 μM of FluB polymerase, purified as described in (8), was mixed with 40 μM of the vRNA 5΄ end (5΄-pAGUAGUAACAAGAG-3΄), 40 μM of vRNA 3΄ end (5΄-UAUACCUCUGCUUCUGCU-3΄) and 40 μM 5΄ capped RNA (m^7^GpppAAUCUAUAAUAGC-3΄) in a buffer containing 500 mM NaCl, 50 mM HEPES pH 7.5, 5% glycerol and 2 mM TCEP. The best diffracting crystals appeared in 100 mM sodium acetate pH 3.8–4.0 and 150 mM di-ammonium phosphate and had a diamond shape. They were frozen with an additional 25% glycerol for cryo-protection. Data were collected on ID23-1 beam line at the ESRF and structure solved by molecular replacement, using the known FluB polymerase structure (PDB 4WRT) (8). Data collection and refinement were done using standard methods and the statistics are given in Table 2.

### Quantifying protein–RNA interactions

Fluorescence spectroscopy was used to quantify protein–RNA interactions, employing either a plate reader or cuvette format. Using the cuvette, protein was successively added to FAM-Ex-5-labelled RNA (if not indicated otherwise) in binding buffer (50 mM HEPES/NaOH, 0.15 M NaCl, 10% (v/v) glycerol, 2 mM Tris(2-carboxyethyl)phosphine (TCEP), pH 7.4) at the concentrations indicated. After attaining equilibrium, total fluorescence intensity and fluorescence anisotropy were measured at *T* = 24°C with a fluorescence spectrometer (Photon Technology International) equipped with polarizers with excitation and emission wavelengths set to 495 and 515 nm, respectively. Total fluorescence intensity was corrected for the volume change due to addition of protein and the observed fluorescence anisotropy corrected for the change in fluorescence intensity according to Equation (1) (17):

(1) \begin{equation*}{f_b} = \frac{{\left( {r - {r_f}} \right)}}{{\left( {\frac{{{I_b}}}{{{I_f}}}} \right)*\left( {{r_b} - r} \right) + r - {r_f}}}\end{equation*}

where *f_b_* represents the fractional concentration of bound RNA, r the observed anisotropy, *r_f_* and *r_b_* the anisotropy of free and bound RNA, and *I_f_* and *I_b_* the fluorescence intensity of free and bound RNA, respectively. Using KaleidaGraph (Synergy Software), dissociation constants were derived by fitting the binding isotherms to a 1:1 binding model of RNA and protein according to Equation (2)

(2) \begin{equation*}{f_b} = \frac{{\left( {L + P + {K_D} - {{\left( {{{\left( {L + P + {K_D}} \right)}^2} - 4*L*P} \right)}^{0.5}}} \right)}}{{2*L}}\end{equation*}

where *f_b_* represents the fractional concentration of bound RNA, *L* the total concentration of labelled RNA, P the concentration of protein and *K_D_* the dissociation constant.

**Figure 1.**
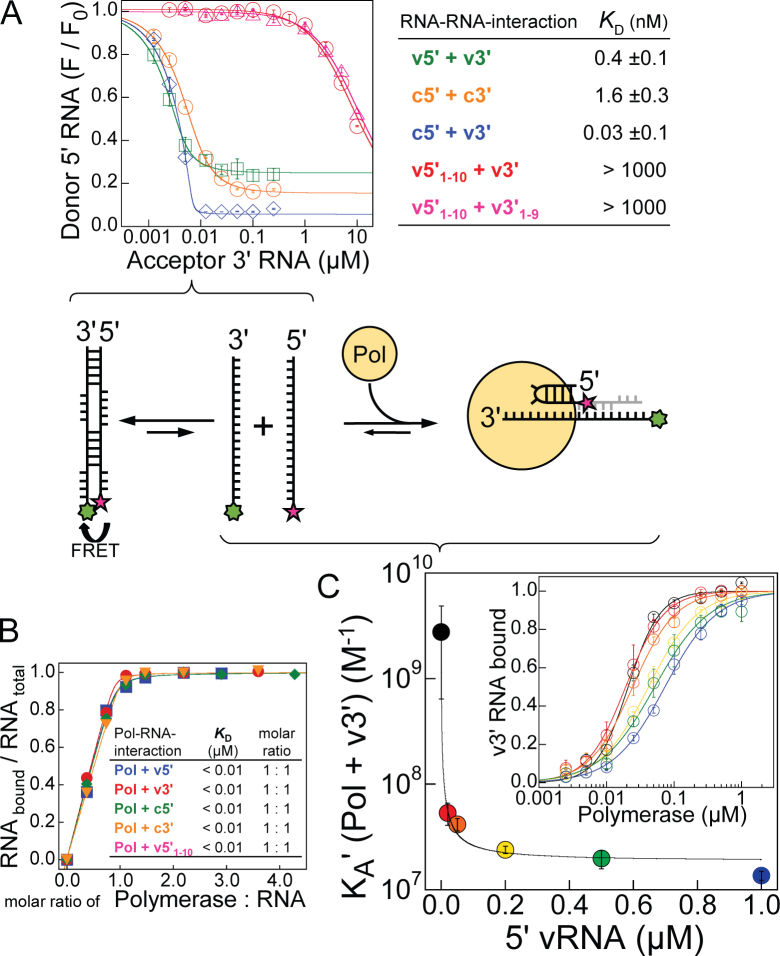
Interaction of influenza polymerase with the promoter. Influenza polymerase binds the promoter 5΄ and 3΄ ends separately and in competition with RNA–RNA intermolecular interactions. (**A**) Due to partial complementarity, the viral 5΄ and 3΄ RNA ends anneal to form a double-stranded ‘panhandle’ conformation as quantified by a simple FRET assay (v5΄, v3΄/c5΄, c3΄ correspond to the genomic/anti-genomic (complementary) 5΄ and 3΄ RNA ends, respectively; all RNAs are 18 nucleotides each if not indicated otherwise; numbering starts from the 5΄ end or 3΄ end of the 5΄ or 3΄ RNA respectively; see Material and Methods). (**B**) Using a fluorescence polarization based assay, the interaction of influenza polymerase and each promoter RNA was quantified and revealed each RNA separately to interact with the polymerase in a one-to-one stoichiometry and with high affinity (*K_D_* < 0.01 μM). (**C**) Strengthening intermolecular RNA duplex conformations by increasing the v5΄ RNA concentration impeded binding of polymerase and the v3΄ RNA with an IC50 = 0.3 nM correlating with the RNA duplex formation. Error bars indicate the standard deviation of the mean of at least duplicate experiments.

Dissociation constants of influenza B polymerase and v3΄ RNA at increasing concentrations of v5΄ RNA were determined in plate-mode format (Figure 1C). Indicated concentrations of influenza B polymerase (pre-incubated with an equimolar concentration of v5΄ RNA nucleotides 1–18) and constant 0.01 μM 5΄-(FAM-Ex-5)-labelled v3΄ RNA (nt 1–18) and 0, 0.02, 0.05, 0.2, 0.5 or 1 μM v5΄ RNA (nt 1–18) were incubated in binding buffer (50 mM HEPES/NaOH, 0.15 M NaCl, 10% (v/v) glycerol, 2 mM Tris(2-carboxyethyl)phosphine (TCEP), pH 7.4) in 384 well plates (FIA plate black, 128.0/85 mm, medium binding; Greiner Bio-One GmbH, Austria) at *T* = 25°C. After attaining equilibrium (∼10 min), fluorescence intensity and polarization were recorded with excitation and emission filters of 485 and 520 nm, respectively (CLARIOstar, BMG Labtech, Germany). Observed fluorescence polarization was corrected for changes in intensity and normalized according to Equation (1) and mean values of independent, at least duplicate experiments plotted with the standard deviation indicated. Binding isotherms were analyzed by the HILL-equation (18). The derived apparent (macroscopic) association constants of influenza B polymerase and the v3΄ RNA were plotted as mean values of at least duplicate experiments (with the standard deviation indicated) against the respective v5΄ RNA concentration and fitted hyperbolically.

### Quantifying RNA–RNA interactions

RNA–RNA interactions were quantified by a simple FRET-assay. Briefly, ∼0.01 μM short 5΄ RNAs labelled at their 3΄ ends by FAM-Ex-5 were incubated at *T* = 25°C in 384 well plates (FIA plate black, 128.0/85 mm, medium binding; Greiner Bio-One GmbH, Austria) in binding buffer (50 mM HEPES/NaOH, 0.15 M NaCl, 10% (v/v) glycerol, 2 mM Tris(2-carboxyethyl)phosphine (TCEP), pH 7.4) with 3΄ RNAs labelled at their 5΄ end by Cy3 at the concentrations indicated. After attaining equilibrium (∼0.5 h), the FAM's fluorescence intensity was recorded as averaged technical triplicates (excitation / emission = 485/520 nm; 10 nm band width; CLARIOstar, BMG Labtech, Germany). The decreasing fluorescence intensity with increasing Cy3-labelled 3΄ RNA is plotted relative to its initial value as mean values of at least duplicate experiments with the standard deviation indicated. Mean values were then analysed according to Equation (2) (with *P* equaling the concentration of Cy3-labelled 3΄ RNA; curve fitting yielded *L* = 0.007 μM and 0.004 μM for FAM-Ex-5-labelled c5΄ and v5΄ nts 1–18, respectively), yielding the corresponding dissociation constants and error of the fitting routine stated. 0.2 μM FAM-Ex-5-labelled v5΄ nt 1–10 were used to study its weak interaction with either Cy3-labelled v3΄ nt 1–9 or v3΄ nt 1–18.

### RNA synthesis assay

Changes of spectroscopic properties associated with FAM-labelled short single-stranded RNA becoming double-stranded are the principle for the new assay monitoring RNA synthesis activity in solution. If not indicated otherwise, 0.25 μM influenza B polymerase pre-incubated with a 1.2 molar excess of v5΄ RNA 1–14 (5΄-pAGUAGUAACAAGAG-3΄) were added to 0.15 μM v3΄ FAM-labelled template RNA (5΄-FAM-Ex-5-UAUACCUCUGCUUCUGCU-3΄) and 0.5 mM NTPs in assay buffer (50 mM HEPES/NaOH, 150 mM NaCl, 10% (v/v) glycerol, 5 mM MgCl_2_, 2 mM TCEP, pH 7.4) at *T* = 24°C to initiate the RNA synthesis reaction. RNA synthesis from the cRNA promoter was performed analogous to the vRNA promoter by using the corresponding cRNA-sequences. To monitor RNA-primed polymerization, reactions were supplemented by 0.5 mM ApG (TriLink BioTechnologies, San Diego, USA or IBA, Goettingen, Germany) or by 1 μM of capped-RNA (5΄-m^7^GpppAAUCUAUAAUAG-3΄; TriLink BioTechnologies, San Diego, USA) if not indicated otherwise. The reactions were incubated in polypropylene reaction tubes and at indicated times aliquots (5 or 10 μl, consistent within one set of reactions) transferred to reaction tubes containing the quenching solution (80 μl 4.5 M NaCl) to reach 4 M NaCl final. After attaining equilibrium, fluorescence polarization (FP) signals were recorded with a CLARIOstar microplate reader (BMG Labtech, Germany) in 384-well plates (FIA plate black, 128.0/85 mm, medium binding; Greiner Bio-One GmbH, Austria) at *T* = 25°C with excitation and emission filters of 485 and 520 nm, respectively. Progress curves were fitted to pseudo-first order reactions according to Equation (3) or to an equivalent double-exponential equation at bi-phasic reaction kinetics:

(3) \begin{equation*}f\ \left( t \right) = \ - A*{e^{ - k*t}} + B\end{equation*}

where *t* is the reaction time, *A* the observed signal amplitude, *k* the observed rate constant and *B* the final FP-signal. The initial reaction rate corresponds to the first derivative of Equation (3) at *t* = 0 and equals *k* times the corresponding fraction of signal-amplitude. The observed signal-amplitude corresponded to the fraction of RNA-product over template RNA. *k*-NTP characteristics for RNA synthesis were fitted according to Equation (4), a simple substrate-inhibition model proposed by HALDANE (19):

(4) \begin{equation*}f\ \left( x \right) = {k_{max}}\ *\frac{x}{{{K_M} + x*\left( {1 + \frac{x}{{{K_i}}}} \right)}}\end{equation*}

where *k*_*max*_ is the maximal rate constant of the RNA-synthesis reaction, *x* the NTP concentration, *K_M_* the Michaelis constant for NTPs and *K_i_* the inhibition constant of NTPs. The *K_M_* of capped RNA primers was determined by Equation (5), the quadratic velocity equation:

(5) \begin{equation*}f\ \left( x \right) = \frac{{\left( {E + x + {K_M} - {{\left( {{{\left( {E + x + {K_M}} \right)}^2} - 4*E*x} \right)}^{0.5}}} \right)}}{{2*E}}\ *A\end{equation*}

with *E* being the concentration of active holoenzyme (polymerase bound to 5΄ RNA and template RNA; *E* = 0.15 μM), *K_M_* the Michaelis constant, *x* the concentration of capped RNA and A the maximal RNA-synthesis reaction.

## RESULTS

### Characterization of influenza polymerase–promoter interactions

To initiate RNA synthesis, influenza polymerase is bound to the promoter which comprises the 5΄ and 3΄ extremities of each (anti-)genome segment and this interaction pseudo-circularizes the vRNA (or cRNA). During initiation, the 3΄ end serves as the template RNA and the 5΄ end as a crucial allosteric co-factor. Due to partial complementarity, the conserved 5΄ and 3΄ extremities in solution can, independently of the polymerase, anneal to adopt the so-called ‘panhandle’ conformation (20–22). *In vitro*, we quantified these RNA–RNA interactions by a fluorescence resonance energy transfer (FRET) assay using short model 5΄ and 3΄ RNAs labelled at their distal ends by fluorescence donor and acceptor, respectively (Figure 1A). The vRNA extremities (v5΄ nt 1–18 and v3΄ nt 1–18) interacted with a *K_D_* ∼ 0.4 nM and a maximal FRET-efficiency (*E*) of ∼0.75 while the corresponding 18-mers of the complementary c5΄ and c3΄ RNAs, the replication intermediate cRNA ends, interacted weaker (*K_D_* ∼ 1.6 nM; *E* ∼ 0.84). Consistent with this, the RNA-folding prediction program Mfold/DINAMelt (23–25) predicts a more stable RNA–RNA interaction between the vRNA ends than the cRNA ends and with a different secondary structure (Supplementary Figure S1). As expected, the perfectly complementary ‘hybrid’ RNA consisting of c5΄ and v3΄ (both nt 1–18) formed the most stable duplex (*K*_*D*_ ∼ 0.03 nM; *E* ∼ 0.94). However, the proximal v5΄ (nt 1–10) and v3΄ (nt 1–9) strands interacted much more weakly under the same experimental conditions (*K_D_* (v5΄ nt 1–10 + v3΄ nt 1–9) = *K_D_* (v5΄ nt 1–10 + v3΄ nt 1–18) > 1 μM). To quantify the interaction of the promoter RNA strands with the polymerase *in vitro*, we used fluorescently-labelled RNAs and measured the change in fluorescence anisotropy upon polymerase promoter complex formation. Influenza B polymerase interacted with each of the 18-mer v3΄, v5΄, c3΄, c5΄ or the v5΄ nt 1–10 separately with a dissociation constant in the low nanomolar range and in a one-to-one stoichiometry (Figure 1B). The v5΄ nt 1–18 impaired the interaction of v3΄ nt 1–18 and the polymerase with an IC50 ∼0.3 nM (Figure 1C), which correlates well with the affinity of the v5΄ and v3΄ to adopt the inter-molecular RNA–RNA ‘panhandle’-conformation (Figure 1A). Using v5΄ and v3΄ RNA variants that showed only weak RNA–RNA interactions (*K_D_* (v3΄ 1–18 + v5΄ 1–10) > 1 μM), a simultaneous binding to the polymerase and in a one-to-one-to-one ratio could be demonstrated (Supplementary Figure S2). These results concur with the published crystal structures where both RNA ends bind to different sites on the polymerase and in competition with the solution ‘panhandle’ conformation (7,8).

### High-throughput compatible influenza polymerase RNA synthesis assay

**Figure 2.**
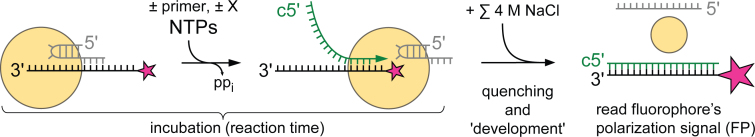
Schematic work flow of the high-throughput compatible RNA synthesis assay. Influenza B polymerase (yellow sphere) activated by 5΄ RNA (nt 1–14) and bound to the 3΄ template RNA (labelled by FAM-Ex-5 at its 5΄ end) is incubated with nucleoside triphosphates (NTPs) and optionally a primer or generally a molecule X whose effect on RNA synthesis is to be monitored. The reactions are quenched by 4 M NaCl final which perturbs polymerase–RNA interactions but permits RNA–RNA interactions. Fluorescence polarization (FP) is recorded after equilibration (development) and directly reads out the ratio of full-length product RNA over labelled template RNA (see Supplementary Figure S3).

An *in vitro* RNA synthesis assay was developed that allows monitoring of the RNA-dependent RNA polymerization activity of influenza polymerase. It is based on two principles: (i) the active polymerase incorporates nucleotides into a nascent product RNA based on complementarity to the template RNA thus potentially producing a template-product duplex; (ii) the fluorescence polarization (FP) of FAM attached to the 5΄ end of the model template RNA changes significantly between its initial single-stranded state and when it is annealed to the complementary product strand. While the total fluorescence intensity essentially remains constant, the observed change in FP displays a direct proportionality to the ratio of complementary product RNA to labelled template RNA at the experimental conditions ([RNA] >> *K_D_* (template + product RNA)) and is sensitive to full-length or full-length-like products only (i.e. in which at least the product end anneals with the end of the template) (Supplementary Figure S3). To facilitate monitoring of the FP changes associated with successful product-formation, the desired FP signal due to duplex formation needs to be de-coupled from that due to labelled template RNA and polymerase interacting. This is achieved by the addition of NaCl to a total of 4 M prior to recording the FP-signal (Supplementary Figure S3). The high ionic strength quenches RNA synthesis and perturbs polymerase–RNA interactions but permits RNA-RNA interactions (Figure 2). At an excess of active polymerase over labelled template RNA, the observed FP increased single-exponentially with progressing reaction time according to pseudo-first order reactions. Fitting single-turnover kinetics (regarding the template RNA) to Equation (3) yielded the corresponding observed rate constants (*k*^obs^), from which the initial rate of RNA synthesis is derived by multiplying *k*^obs^ with the signal-amplitude, in turn proportional to the amount of RNA synthesized. The rate of RNA synthesis showed the expected linear dependency over a wide range of concentrations of active polymerase (i.e. a polymerase bound to the conserved 5΄ and 3΄ extremities of the vRNA), the slope corresponding to the turnover number (*k*_cat_ ∼ 0.25 min^−1^ at 0.5 mM NTPs) (Supplementary Figure S4). This FP-based RNA synthesis assay is not only useful to study the enzymology of influenza polymerase but can also be used to characterize or identify compounds inhibiting RNA synthesis (Supplementary Figure S5). As a proof of principle, similar IC50-values were obtained for example for 2΄F-2΄dCTPs by recording either complete progress curves (at varying compound-concentration) or at only one defined reaction time within the assay's linear range, a criterion for high throughput screening (Table 3; Supplementary Figure S5).

### Transcription versus replication—primed versus unprimed initiation of RNA synthesis

**Figure 3.**
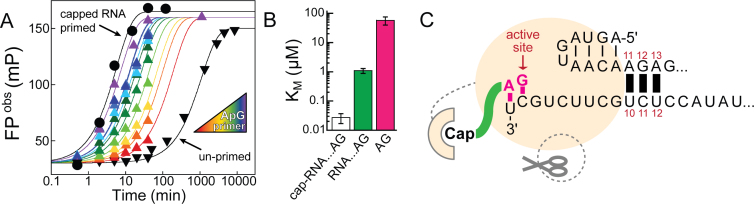
Primed versus unprimed initiation of RNA synthesis by influenza B polymerase *in vitro*. (**A**) ApG, in a concentration-dependent manner (colored triangles), accelerates unprimed RNA synthesis (black triangles) to the level of capped RNA primed RNA synthesis reaction (black circles). (**B**) Affinities (*K_M_*) for the primer(s) in accelerating initiation of RNA synthesis show a preference of the capped RNA over non-capped RNA ending in AG-3΄ over minimally ApG. Error bars indicate the standard deviation of the mean of at least duplicate experiments (see Supplementary Figure S8). (**C**) Schematic depicting the initiation of RNA synthesis catalysed by influenza B polymerase at the vRNA promotor (v5΄ + v3΄). The 5΄-3΄ base-pairing region (involving v5΄ nt 11–13 and v3΄ nt 10–12) positions the 3΄ template RNA at the polymerase's active site. Formation of the first phosphodiester bond (yielding ApG) is rate-limiting for initiating RNA synthesis and bypassed by supplying an appropriate primer. ApG serves as a minimal primer and accelerates unprimed RNA synthesis by annealing to the U1C2 at the 3΄ template extremity. This ‘primed initiation complex’ (PRIC) conformation is further stabilized by favourable interactions of the polymerase with the primer's RNA-moiety and cap-structure.

Within the viral infection cycle, the influenza polymerase initiates RNA synthesis both by primer-dependent and primer-independent mechanisms. Primed initiation of RNA synthesis is employed in cap-dependent transcription, where the polymerase snatches a capped nascent Pol II transcript from the host and trims it to be a capped RNA primer of around 12 nucleotides. The most efficient primers are thought to have ApG at their 3΄ end, allowing them to anneal to the two ultimate nucleotides of the template RNA's 3΄ end thus stabilizing the initiation complex (13,14,26,27). For replication, that is full-length genome amplification via a complementary RNA-intermediate (cRNA), RNA synthesis is initiated *de novo* (unprimed). Employing our new assay, kinetics of both modes of initiating RNA synthesis were recorded with or without a capped 12-mer RNA primer ending in the favored AG-3΄ (Figure 3A). The observed rates of RNA synthesis differed by orders of magnitude (∼ 300; *k*_max primed_ = 0.41 ± 0.013 min^−1^ versus *k*_unprimed_ = 0.0014 ± 0.0006 min^−1^; mean ± SD; *n* ≥ 2), indicating that a rate-limiting step of RNA synthesis is bypassed by providing a suitable primer (Table 1, Figure 3, Supplementary Figures S6–S8). Consistently, ApG accelerated the unprimed reaction to levels of capped RNA primed RNA synthesis reactions in a concentration-dependent manner (Figure 3A and B, Supplementary Figures S6 and S8). Non-capped, 14 nucleotide long RNA ending with AG-3΄ was also able to serve as a primer (Figure 3B, Supplementary Figure S8). The concentration dependence allowed determination of the respective *K_M_*-values (Figure 3B, Supplementary Figure S8) and showed a clear preference for capped RNA (*K_M_* = 0.027 ± 0.01 μM; mean ± SD; *n* ≥ 2). By comparing this result with the *K_M_* of non-capped RNA (*K_M_* = 1.1 ± 0.2 μM; mean ± SD; *n* ≥ 2) and ApG alone (*K_M_* = 58 ±18 μM; mean ±SD; *n* ≥ 2), it is clear that both the cap and the RNA moiety of the primer contribute to enhance efficiency. To obtain insights into the enthalpic and entropic contributions of the initiation of RNA synthesis, the unprimed and primed reactions were performed at different temperatures (Supplementary Figure S6). An Arrhenius analysis revealed that both reactions require the same activation energy of *E_A_* ∼ 50 kJ/mol but differ in the entropic term, which can be related to the frequency of reacting particles colliding. All reactions were performed under single-turnover conditions regarding the template RNA (v3΄ 1–18 nt) and with the polymerase activated by the v5΄ 1–14. Shortening the v3΄ template RNA below 12 nucleotides or truncating the v5΄ to nt 1–12 or shorter resulted in inactivation of RNA synthesis (Supplementary Figure S9), highlighting the importance of the base-pairing region between v3΄ and v5΄ indicated in the scheme (Figure 3C).

**Table 1.** Enzymatic parameters for influenza B polymerase initiating RNA synthesis from the vRNA or cRNA promoter *in vitro*

	*K_M_* (capped RNA) (μM)	*K_M_* (NTPs) (mM)	*k*_*cat*_ (min^-1^)	*k*_*cat*_/(K_M_*K_M_) (min^-1^ μM^-1^ mM^-1^)	*k*_*de novo*_ (min^-1^)
vPol	0.027 ± 0.01	0.037 ± 0.005	0.41 ± 0.013	∼410	0.0014 ± 0.0006
cPol	0.12 ± 0.03	0.32 ± 0.11	0.10 ± 0.004	∼2.6	0.0006 ± 0.0001

*K_M_* (capped RNA) was determined by the dependency of the rate of RNA synthesis (mP/min) on the concentration of capped RNA primer at otherwise saturating conditions (0.5 mM NTPs, all template RNA (0.15 μM) bound by polymerase (0.25 μM); see Materials and Methods and Supplementary Figure S8). *K_M_* (NTPs) and *k*_*cat*_ were determined by analyzing the effect of varying the NTP-concentration on capped RNA primed RNA synthesis according to a simple substrate-inhibition model (19,58) at otherwise saturating reaction conditions (Materials and Methods, Figure 6A, Supplementary Figure S10). The catalytic efficiency of capped-RNA primed RNA synthesis is expressed as *k*_*cat*_/[*K_M_* (NTPs) x *K_M_* (capped RNA)]. The rate constant of unprimed (*de novo*) initiation of RNA synthesis, *k*_*de novo*_, was determined at 0.25 ±0.05 μM influenza B polymerase and at 0.5 mM NTPs (each) (see Supplementary Figure S7). All experiments were performed in assay buffer at *T* = 24°C with polymerase activated by a 1.2-fold excess of v5΄ 1–14 or c5΄ 1–14, respectively and 0.15 μM fluorescently-labelled template RNA v3΄ 1–18 or c3΄ 1–18, respectively (see Supplementary Table S1). Stated values are the mean of at least duplicate experiments with the corresponding standard deviation.

### RNA synthesis from the vRNA and cRNA promoters differ enzymatically

Replication of the viral genome requires that the influenza polymerase initiates RNA synthesis at the vRNA and cRNA promoter to produce cRNA and vRNA, respectively. Structural analysis has shown that the vRNA promoter consists of the conserved 5΄ and 3΄ extremities with 5΄ nucleotides 1–10 adopting a special hook-structure anchoring it to the polymerase and nucleotides 11–14/15 forming a base-pairing region with nucleotides 10–13/14 of the 3΄ template RNA (Figure 3C) (7,8). This conformation positions the v3΄ template RNA at the active site for terminal initiation. Based on the high complementarity of vRNA and cRNA, the cRNA promoter is likely to adopt a similar conformation. Indeed, the c5΄ has been observed to adopt the same hook structure and undistinguishable binding to the polymerase (28) (Figure 1). However under identical conditions, influenza polymerase initiating RNA synthesis at the vRNA promoter (vPol), compared to the cRNA promoter (cPol), displayed around a four times improved apparent rate constant, about nine times improved *K_M_* for NTPs and about five times improved *K_M_* for the capped RNA primer (Table 1, Figure 6A, Supplementary Figures S8 and S10), resulting in an ∼200-fold higher catalytic efficiency of vPol over cPol for initiating capped RNA primed RNA synthesis.

### vPol versus cPol—terminal versus internal initiation of RNA synthesis

**Figure 4.**
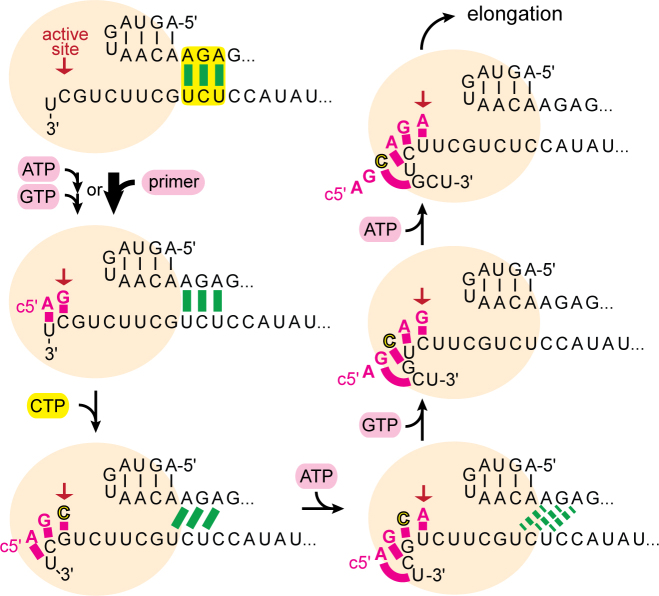
Schematic model of influenza polymerase initiating RNA synthesis at the vRNA promoter. The 5΄-RNA is firmly anchored to the polymerase (beige sphere), the 3΄ template RNA positioned for terminal initiation at the active site by intermolecular interactions of the distal promoter RNAs (5΄-3΄ base-pairing region; highlighted, green base-pairs). Simultaneous binding of ATP at the post-translocation site and GTP at the active site (pre-translocation) followed by formation of the first phosphodiester bond is bypassed by an appropriate primer ending in AG-3΄. CTP, the next nucleotide to be incorporated and directed by G3 on the vRNA template is the first (and within the five ultimate nucleotides the only) distinction from the cRNA template. To allow for translocation of the template RNA and continued RNA synthesis (elongation), the 5΄-3΄ base-pairing region must dissipate which we propose is coupled to the newly emerging base-pairs between product and template RNA downstream the active site (pink). At the cRNA promoter, terminal fast initiation of RNA synthesis is prevented since with UTP directed to position A3, an A–U base pair is formed, instead of the G–C base-pair in the case of vRNA, which apparently does not compensate for breaking the cRNA promoter's 5΄-3΄ base-pairing region. At the expense of rate, the cRNA promoter re-directs RNA synthesis via internal initiation followed by re-align (see Figure 5).

**Figure 5.**
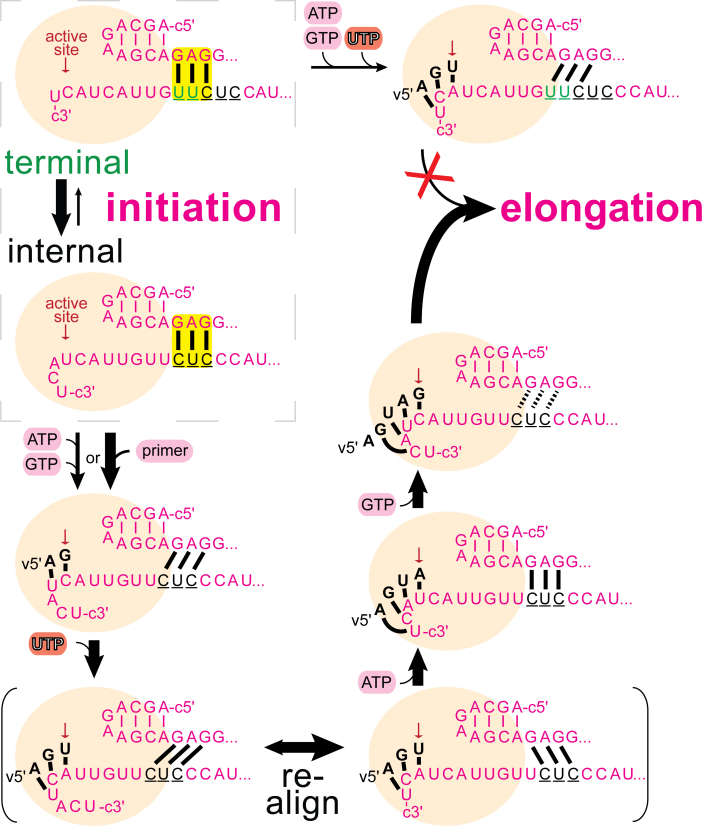
Schematic and hypothetical model of internal initiation of RNA synthesis at the cRNA promoter involving *prime-and-re-align*. Influenza B polymerase is depicted by a beige sphere with the location of the active site indicated by the arrow. The c5΄ end (pink) is firmly anchored to the polymerase, and helps position the c3΄ 1–18 template RNA (pink) for terminal or internal initiation at the active site. The depicted positioning of the c3΄ template RNA for internal initiation would be stabilized by a 5΄-3΄ base-pairing region involving (at least) c5΄ nucleotides G11, A12 and G13 and c3΄ nucleotides C12, U13, C14, in analogy to the vRNA promoter. The initial ATP would bind opposite of U4 at the active site in the pre-translocation conformation. Template RNA translocation by one step places the initial ATP into the post-translocation state, allowing binding of incoming GTP opposite to C5 at the active site and its incorporation to form pppApG. With the template RNA having translocated by 1 position, strain in the 5΄-3΄ base-pairing region would be generated. Further translocation of the template RNA and pppApG to the post-translocation conformation allows binding of incoming UTP opposite of A6 at the active site and its subsequent incorporation. However, this would build-up additional unfavourable strain in the 5΄-3΄ base-pairing region which would need to be relieved. However, breaking the strong 5΄-3΄ base-pairing region formed by the cRNA promoter (2x GC, 1x AU) is not the most favorable outcome at this stage since the newly formed base-pairs between the product RNA and the template RNA (2x AU, 1x GC) downstream of the active site do not compensate energetically. More favorably, the template RNA would translocate three steps backward while the product RNA stays in place. This alternative position of the template would then promote incorporation of the next nucleotide, through binding of ATP opposite of U4, as this restores three unstrained base-pairs in an alternative 5΄-3΄ base-pairing region. Translocation, binding of GTP opposite of C5 and formation of pppApGpUpApG would again build-up strain in the 5΄-3΄ base-pairing region. However, at this stage more newly formed base-pairs between the product RNA and the template RNA are available (compared to having initiated terminally) which would compensate for breaking the 5΄-3΄ base-pairing region and allow RNA synthesis to progress to elongation.

**Figure 6.**
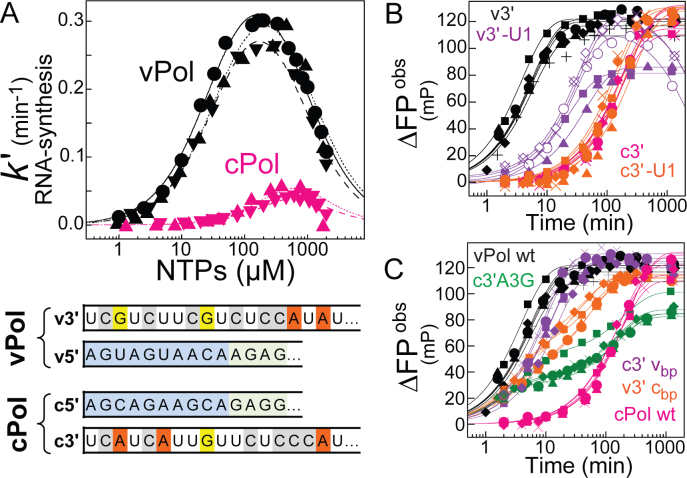
Terminal (vPol) versus internal (cPol) initiation of RNA synthesis. (**A**) The vRNA promoter (vPol; black; 3 independent rate-NTP-characteristics, circles, upward and downward triangles) or the cRNA promoter (cPol; pink; 2 independent rate-NTP-characteristics, upward and downward triangles) direct the initiation of RNA synthesis by influenza B polymerase with different enzymatic parameters (Table 1). The dependency of RNA synthesis (observed rate constants) on the substrate-concentration (NTP) was fitted according to a simple substrate-inhibition model proposed by HALDANE (19) (see Materials and Methods). The promoter RNA sequences responsible for the altered *k*_*cat*_, *K_M_* (NTPs) and *K_M_* (primer) are shown in the lower panel. Both the v5΄ and the c5΄ comprise a proximal hook structure (nt 1–10; blue colouring) followed by the distal 5΄-3΄ base-pairing region (nts 11–13/14; green colouring). The v3΄ and c3΄ template RNAs each direct the incorporation of NTPs based on complementarity. (**B**) Deletion of the ultimate U1 from the template RNA impairs initiation of RNA synthesis from the vRNA promoter but not cRNA promoter, indicating terminal and internal initiation mechanisms, respectively. Shown are five independent progress curves each for initiation at the vRNA and cRNA promoter (black and pink) and the corresponding U1 deletions, respectively (purple and orange). (**C**) vPol-like kinetics (black) were recovered by a cPol with either (i) A3 of c3΄ substituted by G3, as found in vRNA (c3΄A3G; green); or (ii) the distal cRNA promoter involving the 5΄-3΄ base-pairing region being swapped for the corresponding region of the vRNA promoter (5΄ nt 11–18, 3΄ nt 10–18; c3΄ v_bp_, purple); or (iii) the proximal cRNA promoter swapped for the corresponding region of the vRNA promoter (5΄ nt 1–10, 3΄ nt 1–9; v3΄ c_bp_, orange) (see Supplementary Figure S12). Only the c3΄ template RNA in combination with the cRNA promoter's 5΄-3΄ base-pairing region resulted in cPol-like slow progress curves indicating internal initiation (pink). The bi-phasic kinetics of RNA synthesis by some RNA combinations was attributed to strengthened RNA–RNA interactions which kinetically competed with polymerase for single-stranded template RNA (Figure 1, Supplementary Figure S13). In each case, five independent kinetics are shown.

Different *de novo* replication initiation mechanisms have been proposed for vPol and cPol (16,29). Whereas vPol was shown to initiate RNA synthesis terminally at U1/C2 (Figure 4), cPol was demonstrated to initiate internally at U4/C5 followed by a re-alignment of the short product RNA with respect to the template RNA before resuming RNA synthesis (Figure 5). In agreement, we observe that deleting U1 from the vRNA promoter's template RNA reduced the rate constant of RNA synthesis from *k* = 0.19 ± 0.03 min^−1^ to *k* = 0.03 ± 0.01 min^−1^ (mean ± SD; *n* = 5; Figure 6B). RNA synthesis from the cRNA promoter was not impaired by the corresponding deletion of c3΄s U1 (Figure 6B). Interestingly, the effect of the template's ultimate U1 was conserved in both primed and unprimed initiation of RNA synthesis and thus is independent of the priming-mechanism (Supplementary Figure S11). By providing an appropriate primer, the initiation of RNA synthesis was de-coupled from the rate-limiting ApG-formation but the mechanism of initiation remained conserved: fast for terminal initiation by vPol and slow for internal initiation by cPol (*k* = 0.19 ± 0.03 min^−1^ and *k* = 0.0055 ± 0.001 min^−1^ (mean ± SD; *n* = 5), respectively at capped RNA primed conditions and 0.025 mM NTPs, Figure 6B). Since only the RNA-sequences differed in these reactions, there must be some specific information encoded in them that determines the mode of initiation of RNA synthesis. By swapping RNA-elements within the vRNA and cRNA promoters and measuring the effect on RNA synthesis, two determinants were found to direct fast, terminal or slow, internal initiation of RNA synthesis (Figure 6C). vPol-like kinetics were recovered by a modified cPol promoter with either (i) A3 of c3΄ substituted by the v3΄ corresponding G3 (c3΄A3G; *k* = 0.47 ± 0.12 min^−1^ (mean ± SD; *n* = 5)); or (ii) the distal 5΄-3΄ base-pairing region of the cRNA promoter being swapped for the corresponding vRNA promoter (5΄ nt 11–18, 3΄ nt 10–18; c3΄v_bp_; *k* = 0.09 ± 0.01 min^−1^ (mean ± SD; *n* = 5)). In addition, swapping of the proximal cRNA promoter RNAs for the corresponding vRNA promoter (5΄ nt 1–10, 3΄ nt 1–9; v3΄c_bp_; *k* = 0.29 ± 0.04 min^−1^ (mean ± SD; *n* = 5)) (Supplementary Figure S12). Only the c3΄ template RNA in combination with the cRNA promoter's 5΄-3΄ base-pairing region resulted in cPol-like slow kinetics indicative of internal initiation. Note that the bi-phasic progress curves observed with a promoter consisting of a vPol-like proximal (3΄ nt 1–9; 5΄ nt 1–10) and cPol-like distal (3΄ nt 10–18; 5΄ nt 11–18) part or with just the c3΄ A3G variant of the cPol did not necessarily reflect the proposed heterogeneous positioning of the template RNA (Figure 5). Rather, the first phase proceeded with vPol-like reaction rates while the second phase was dependent on the intermolecular interactions of the promoter RNAs that kinetically competed with and impeded RNA synthesis (see Supplementary Figure S13). Briefly, similarly stable intermolecular RNA–RNA interactions are predicted for e.g. the chimeric vRNA promoter but with the c5΄ hook (nt 1–10) or the chimeric cRNA-promoter but with the v5΄ hook (nt 1–10) (*T_m_* = 54°C and 51°C, respectively; see Supplementary Figure S13). However, only for the former chimeric vRNA-promoter, vPol-like RNA synthesis, but with reduced amplitude, was observed. No RNA synthesis was detected with the chimeric cRNA-promoter which directs for slow RNA synthesis. This argues for a kinetic competition of the RNA synthesis, which requires a single-stranded and accessible template RNA, with the annealing of the two promoter RNAs. The origin of the 5΄ RNA hook structure (nt 1–10, cRNA or vRNA) did not show a detectable effect on initiation (Supplementary Figure S13).

### Energetic compensation through breaking and making of base-pairs during initiation of RNA synthesis

Since both the 5΄-3΄ base-pairing region and the template-directed first few base-pairs are responsible for fast, terminal or slow, internal initiation, we propose that breaking promoter base-pairs is energetically compensated by making new template-product base-pairs in the active site (Figures 4 and 5). To couple translocation of the template RNA with product RNA formation, the distal 5΄-3΄ base-pairing region of the promoter, that initially positions the template RNA at the active site, needs to be resolved. Our data are consistent with the newly formed, temporary base-pairs downstream of the active site (closer to the template exit) compensate with the breaking the 5΄-3΄ promoter base-pairing region, which is essential for proceeding to elongation. When RNA synthesis initiates terminally, the first three nucleotides directed by the v3΄ template RNA would form 1 AU and 2 GC base-pairs which would energetically compensate breaking the two AU and one GC of the vPol-like 5΄-3΄ base-pairing region formed between 5΄ nt 11–13 and 3΄ nt 10–12. For cPol, the promoter RNA-sequences allow a heterogeneous positioning of the c3΄ template RNA for both terminal and internal initiation (Figure 5). In the native cRNA promoter, terminal initiation is prevented and re-directed via the slower, internal initiation mechanism involving prime-and-re-align (Figure 5 and Discussion). Terminal initiation by cPol becomes favorable for example when A3 is substituted by G3 (as in vPol) (Figure 6C) which results in the exchange of an AU- for a GC-base-pair between the template and the emerging product.

### Crystal structure of influenza B polymerase with 3΄ end of vRNA in the active site

**Figure 7.**
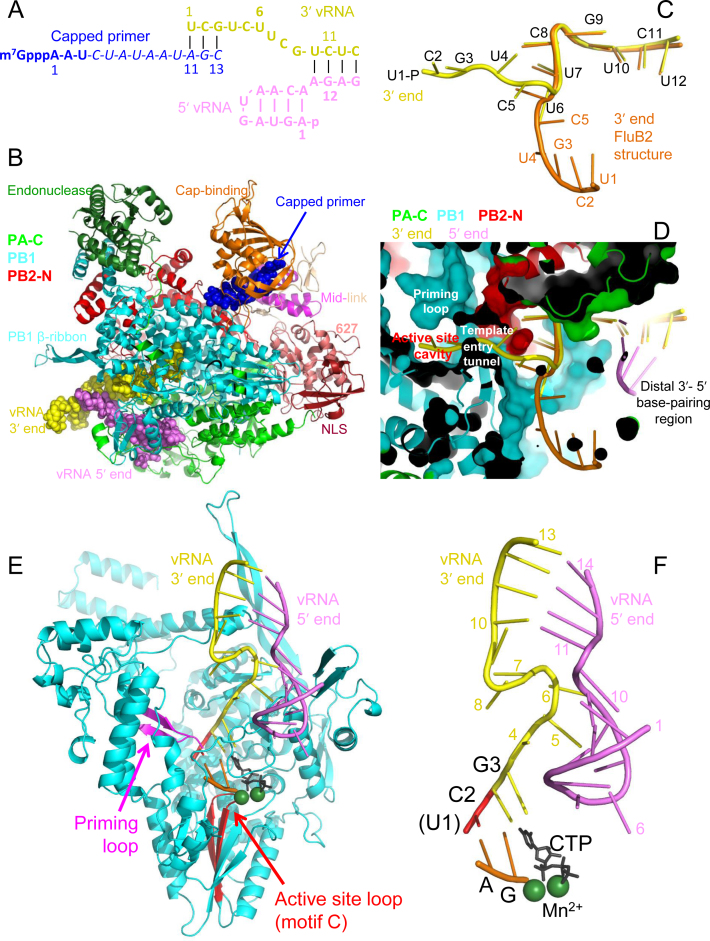
Structure of influenza B polymerase co-crystallized with the vRNA promoter and capped primer. (**A**) Schematic of RNAs used for crystallization of the putative transcription initiation state. Nucleotides 4–13 of the 13-mer capped RNA primer are in italics as they are not seen in the structure. (**B**) Overall view of the crystal structure with influenza B polymerase in ribbon representation colored according to domains as indicated. The RNA in space-filling representation with the capped primer (blue), 5΄ end (violet) and 3΄ end (yellow), as in (A). (**C**) Trajectory of the 3΄ end (template) as observed in the current structure (yellow) and in the previous FluB2 structure (orange) (8). (**D**) As (C) but in the context of the polymerase (surface and ribbon representation) showing the template passing through the entry tunnel into the active site cavity. (**E**) Overall view of the promoter bound to the PB1 subunit (cyan ribbons) showing the priming loop (purple) and active site motif C (red). The unobserved 3΄ terminal base (U1, red) is modeled. Based on superposition with the Norwalk virus polymerase primer-template/substrate (CTP) complex (PDB: 3BSO, (30)) the initial ApG of the product (orange), the incoming CTP (black) and the two catalytic divalent ions (green spheres) are modelled. (**F**) As (E) but only showing the RNA.

In previous structures of influenza B polymerase with bound promoter the 3΄ extremity (nt 1–6) was not observed in the internal active site cavity but was either not visible, due to disorder (FluB1 crystal form) or bound on the polymerase surface (FluB2 crystal form) (8). We attempted to determine a crystal structure of the cap-dependent transcription initiation state by co-crystallizing influenza B polymerase with the vRNA promoter (5΄ nt 1- 14, 3΄ nt 1- 18) and a capped 13-mer primer ending in AGC-3΄, which could in principle make three base-pairs with the terminal 3΄-UCG of the template (Figure 7A). A trigonal crystal form, similar to form FluB1 but grown at lower pH (see Materials and Methods), yielded a structure at 3.8 Å resolution ( Table 2), which, for the first time, clearly shows the 3΄ extremity in the active site (Figure 7A and B, Supplementary Figure S14A). However the electron density for 3΄ nucleotides 1–6 is weaker than that for nucleotides 7–18 indicating partial occupancy. This could be due either to 3΄ RNA degradation or a mixture of conformations between the disordered surface configuration (previously observed in form FluB1) or entering the active site. Of the capped primer, only the first few nucleotides (m7GpppAAU) are visible in the electron density bound to the PB2 cap-binding and mid-domains (Figure 7B) but the expected extension of the primer to base-pair with the 3΄ extremity of the template in the active does not occur in this crystal form. Apart from this, the FluB polymerase structure is essentially identical to that previously described for form B1 (8), notably the base-paired configuration of the distal part of the promoter and the hook conformation of the 5΄ end. The structure shows that flexibility at nucleotide U6 allows the proximal 3΄ end to be either outside the polymerase (as in previous structures) or pass through the template entrance tunnel into the active site cavity (Figure 7C and D). Remarkably, only small side-chain movements (e.g. PB2 Arg40, Lys43; PB1 Met227 and the Motif B loop 409–410) allow the 3΄ end to thread through the narrow template entry tunnel. Although the detailed interactions of the template with the entry tunnel cannot be elucidated with certainty at the current resolution, the electron density suggests that highly conserved residues from the anti-parallel β-strands at the base of the fingertips loop and from the N-terminus of PB2 are involved (Supplementary Figure S14B). Only the phosphate of U1 has electron density, the rest of the nucleotide being disordered as well as the adjacent tip of the priming loop (646-PAHG-649 lack density). Superposition of the polymerase active site with that of Norwalk virus polymerase primer–template/substrate (CTP) complex (PDB: 3BSO, (30)) suggests that the 3΄ extremity is positioned not for terminal initiation but advanced one position (Figure 7E and F). Three base-pairs could potentially be made in the active site, with G3 opposite to the incoming NTP position. However in the structure, neither the end of the co-crystallized primer, nor a soaked in ApG dinucleotide are observed making such base-pairs in the active site. This suggests that an additional conformational change is required to generate the active initiation state and this might be hindered by the strong constraints on the duplex region of the promoter imposed by the intermolecular RNA–RNA crystal contacts (8). Nevertheless, the structure shows that even without breaking the base-paired region of the promoter the 3΄ end of the vRNA is able to reach the active site. Secondly, it suggests that the structure of the cRNA promoter must differ from that of the vRNA promoter so that the 3΄ end can reach further into the active site cavity and allow internal initiation. This could be achieved for instance by an alternative 3΄-5΄ base-pairing scheme involving 3΄ nt 12–14 (instead of 10–12) with 5΄ nt 11–13 (Figure 5).

**Table 2.** Crystallographic data collection and refinement statistics

**Crystal**	FluB polymerase
	+3΄ vRNA 1–18
	+5΄ vRNA 1–14
	+13-mer capped RNA primer
	
**Diffraction data**	
Space group	*P*3_1_21
Cell dimensions (Å)	*a* = *b* = 200.4
	*c* = 254.6
	α = β = 90, γ = 120
Wavelength (Å)	1.2724
Beamline	ID23-1 (ESRF)
No. of crystals	3
Resolution range (last shell) (Å)	50.0-3.8 (4.0-3.8)
Completeness (last shell) (%)	99.9 (100)
*R*-sym (last shell)	0.411 (2.74)
*I/σI* (last shell)	9.76 (1.20)
Redundancy (last shell)	46.4 (45.1)
**Refinement**	
Reflections in refinement: work (free)	55 900 (2785)
*R*-work (last shell)	0.235 (0.424)
*R*-free (last shell)	0.267 (0.397)
Number of non-hydrogen atoms	18236
Protein	17457
RNA	769
Solvent	10 (2 x phosphate)
**Geometry**	
RMS (bonds)	0.007
RMS (angles)	0.996
Ramachandran favored (%)	93.5
Ramachandran outliers (%)	0.4
Molprobity score	1.59
Clash score	1.41
**Average *B*-factors** (Å^2^)	
All atoms	177.0
Protein	177.1
vRNA 5΄, 3΄	142.2, 182.0
Capped RNA	244.1

### Initiation by vPol and cPol is targeted differently by 2΄F-2΄dNTPs

Using *in vitro* and cell culture experiments, 2΄F-2΄dGTP had been established to inhibit influenza polymerase reversibly and competitively with respect to GTP (31). By determining the IC50-profiles for each of the four 2΄F-2΄dNTPs at 0.025 mM and 0.25 mM NTPs, we showed that all four 2΄F-2΄dNTPs competitively inhibit influenza polymerase initiating RNA synthesis at the vRNA promoter, with respect to NTPs (Table 3, Supplementary Figure S5). The purines 2΄F-2΄dATP and 2΄F-2΄dGTP were found to be more potent compared to the pyrimidines 2΄F-2΄dCTP and 2΄F-2΄dUTP (IC50-values at 0.025 mM NTPs of 0.03 mM and 0.02 mM versus 0.21 and 0.45 mM, respectively). This could be due to potential additional contributions of the purine- over the pyrimidine-moiety or due to their proportionally high demand directed by the template RNA involved or some other effect (Table 3, see panel Figure 6A). Initiation of RNA synthesis from the cRNA promoter was also inhibited by 2΄F-2΄dNTPs, but rather non-competitively with respect to NTPs, unexpectedly and in marked contrast to vPol (Table 3). An explanation could be the existence of an alternative site besides the active site (e.g. incoming NTP; pre-, post-translocation; see (32)), more accessible in cPol for the sugar-modified nucleoside-analogues probed. Substituting just the 5΄ hook structure of the vRNA promoter for the corresponding c5΄ nucleotides 1–10 maintained the vPol-like inhibition profiles, showing again that the origin of the 5΄ hook is irrelevant for initiating of RNA synthesis (Table 3). Substituting just the distal 5΄ and 3΄ RNAs (comprising the 5΄-3΄ base-pairing region) for the corresponding cRNAs again yielded vPol-like IC50-values and vPol-like competitiveness with respect to NTPs (Table 3). Only the combination of a c3΄-like template RNA (A3) with a cPol-like 5΄-3΄ base-pairing region, as required to direct initiation of RNA synthesis via internal initiation and re-alignment, resulted in this special mechanism of inhibiting initiation of RNA synthesis at the cRNA promoter by 2΄F-2΄dNTPs.

**Table 3.** Inhibition profiles of 2΄F-2΄dNTPs for influenza B polymerase initiating RNA synthesis at different RNA promoters

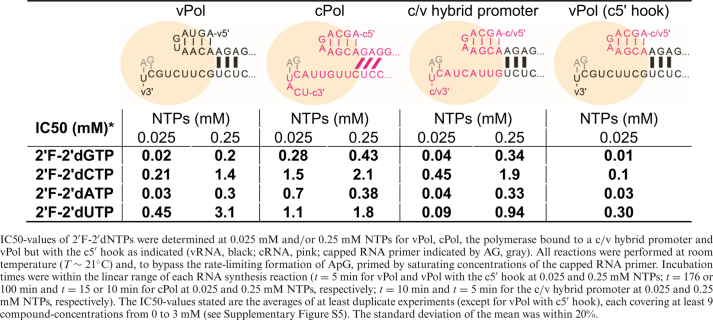

### Early initiation is more susceptible to inhibition by 2΄F-2΄dNTPs

During initiation of RNA synthesis, influenza A polymerase in the context of a native RNP produces small abortive transcripts of a capped RNA (ending with … AG-3΄) elongated by only one or, less efficiently, by two nucleotides (33). Only subsequently does it switch to a more processive state referred to as elongation. Since the enzymatic properties of influenza polymerase were demonstrated to be altered by this switch (33), the susceptibility to inhibition might also be affected. As a simple proof of principle, we compared the IC50-profiles of particularly 2΄F-2΄dCTP and 2΄F-2΄dUTP for vPol and the polymerase bound to the chimeric promoter RNA of cRNA proximal and vRNA distal parts (c/v hybrid promoter; Table 3). Both these polymerase–promoter complexes were shown to initiate RNA synthesis terminally, fast and with vPol-like inhibition profiles (competitiveness of 2΄F-2΄dNTPs and NTPs). However, they differ in the template directed requirement for incorporating CTP and UTP at certain positions (see Figure 6A, lower panel and Table 3, compare ‘vPol’ and ‘c/v hybrid promoter’). In vPol, 2΄F-2΄dUTP is directed first to position 15. With the c/v hybrid promoter, 2΄F-2΄dUTP is first directed by the template to position 3 which improved the IC50 (2΄F-2΄dUTP) from 0.45 to 0.09 mM. Reversely and consistently, the IC50 for 2΄F-2΄dCTP of 0.2 mM with vPol, which directs it to position 3, dropped to 0.45 mM with the c/vRNA hybrid promoter where CTP is first directed to position 9. We did not study additional more artificial RNA-variants because of the high conservation of the RNA-sequences and because it is difficult to exclude other effects of nucleotide-substitutions (e.g. effecting the equilibrium between promoter binding to the polymerase or forming a double-stranded ‘panhandle’-conformation), as also noted by others (34). However, our results provide clear evidence that early initiation is more susceptible to inhibition by 2΄F-2΄dNTPs than subsequent stages of RNA synthesis.

## DISCUSSION

Fluorescence polarization (FP) is widely used to study protein-nucleic acid-interactions (17,35,36). From the change of FP associated with complex formation between a short, fluorescently labelled nucleic acid and a protein, equilibrium constants and the stoichiometry of interaction can be derived (37). However, hybridization of a fluorescently labelled oligonucleotide to its complementary unlabelled oligonucleotide also gives an increased FP signal as shown for HEX, rhodamine (38) and FAM-Ex-5 (this study). Based on this phenomenon we developed an assay that directly reports influenza polymerase product-formation, thus expanding the application of fluorescence techniques to quantify RNA synthesis *in vitro* (36,39,40). It should be noted that influenza polymerase likely directs template and product strands out through distinct tunnels specifically to avoid extensive duplex formation (41), hence the need to quench the reaction in a way that dissociates the RNA from the polymerase. This method is clearly applicable to other polymerases since they all function to synthesize a complementary strand to a given template nucleic acid. Assuming the polymerase accepts fluorophore-labelled templates, quenching of the reaction might require minor adjustments, although for the polymerases of HCV (42,43), poliovirus (39,44,45) and *Escherichia coli* RNA polymerase (46), a high ionic strength has been demonstrated to perturb their interaction with nucleic acids. The assay monitors the formation of double-stranded nucleic acids, but could be adapted to follow the reverse reaction allowing study of helicases and nucleases for example or possibly nucleic acid chaperones and nucleic-acid folding. The FP-based assay provides an attractive alternative to the conventional RNA synthesis assay that monitors the template-directed incorporation of radio-labelled nucleotides into acid-insoluble products (47). Indeed, we have verified that the two assays give essentially the same kinetic parameters under the same conditions (Supplementary Figure S15). Whilst the separation of RNA-products via gel-electrophoresis prior to phosphor-imaging yields important additional information about the products’ size, assays using radioactivity involve time- and labor-intense handling of toxic material. In contrast, the solution-based FP assay described here reports predominantly on full-length products and is less suited to detect smaller abortive products. This is because the fluorescent probe that is sensitive to changes of its environment is located at the 5΄ end of the template RNA and the polymerase translocates on the template RNA with 3΄-5΄ directionality.

Knowledge of enzymatic parameters for influenza B polymerase, such as the affinity for the template RNA, crucial co-factors (v5΄ extremity) and substrate NTPs, as well as catalytic rates for the differently primed RNA synthesis reactions, were important for development of an optimized and robust screening assay. Briefly, maintaining an excess of primer over active polymerase over template RNA at concentrations exceeding the *K_D_* (*K_M_*), pseudo first-order conditions are fulfilled and the reaction progresses single-exponentially. Determining the FP-signal at one defined reaction time within linear range of the assay is sufficient for preliminary screening of compound libraries. As a proof of principle, we demonstrated the assay's HTS-capabilities by characterizing a set of nucleoside-analogues for their potential to inhibit RNA synthesis (Table 3, Supplementary Figure S5).

The *in vitro* enzymatic parameters of influenza B polymerase that we measured (Table 1) now need to be placed in the physiological context. We find that vPol (polymerase associated with the vRNA promoter) is ∼200-fold more efficient in transcription initiation than cPol (polymerase associated with the cRNA promoter). That this is not due to a slower assembly of cPol was shown by pre-incubating polymerase and the cRNA promoter (Supplementary Figure S16). vPol not only had an improved catalytic rate (*k*_*cat*_) but also higher affinity for the substrate NTP (*K_M_*) and the capped RNA primer (Table 1). This is consistent with the fact that in the infected cell vRNPs are effectively the only functional unit producing viral mRNA transcripts. However, since transcription can only occur if the RNP has access to capped primers, this might be an additional factor limiting formation of anti-sense mRNAs by cRNPs (in any case these would not be correctly poly-adenylated) (see below). Since the promoter RNA sequences are the only variable in this comparison of vPol with cPol, the differences in behavior must depend on these sequence differences and corresponding structural changes they might induce. Unfortunately, there is still no structure of influenza polymerase binding the full cRNA promoter which might give insight into the difference between vPol and cPol. However, our data, particularly the effect of omitting U1 from the template RNA, are in agreement with previous reports (16) that there are different initiation mechanisms for vPol and cPol, terminal at U1/C2 and internal at U4/C5, respectively. Additionally, we correlate the vPol-like terminal initiation mechanism with fast (∼0.2 min^−1^) and the cPol-like internal initiation mechanism involving *prime-and-re-align* with slow rates (∼0.006 min^−1^) *in vitro*. The crystal structure of influenza polymerase bound to the vRNA promoter in the pre-initiation conformation shows a 5΄-3΄ base-pairing region in the distal promoter region involving 5΄ nucleotides 11–13 base-pairing with 3΄ nucleotides 10–12 (7,8). In the new structure reported here (Figure 7), the promoter base-pairing is preserved and the 3΄ end is observed, for the first time, entering the active site cavity although base-pairing with the end of the co-crystallized capped primer is not observed. The 5΄-3΄ base-pairing region is essential for initiation of RNA synthesis (Supplementary Figure S9), presumably because it serves to position the template 3΄ end correctly in the active site. However, the base-pairs must subsequently be broken to allow template translocation and product elongation, although exactly how and at what step this occurs is still unclear. Supported by experimental evidence (Figure 6), we propose a model for terminal initiation of RNA synthesis (Figure 4) whereby the energy cost of breaking the promoter 5΄-3΄ base pair region is compensated by that gained when new base-pairs are formed between the template RNA and the product RNA downstream of the active site (referred to as the *initiation signal*). It remains to be seen what degree of mechanistic coupling exists between the making and breaking of these compensating base-pairs. Only the wild-type cPol promoter with its combination of a strong 5΄-3΄ base pairing region (consisting of 1G:U, 1G:C, 1 A:U) and a weak initiation signal (yielding sequentially A:U, G:C, U:A) resulted in slow, internal initiation (Figures 5 and 6). Mutating the cPol promoter to weaken the 5΄-3΄ base pairing region to the situation in vPol (2 A:U, 1 G:C) or to strengthen the initiation signal to the vPol-like A:U, G:C, C:G resulted in fast vPol-like RNA synthesis rates via terminal initiation. In agreement with the *prime-and-re-align* model of internal initiation (16), RNA synthesis initiating at U4/C5 would be blocked from directly proceeding to elongation by the strong 5΄-3΄ base pairing region formed by 2 G:C and 1 A:U bp between c5΄ nucleotides 11–13 and c3΄ nucleotides 12–14 that stabilized the template RNA positioned for internal initiation (Figure 5). After initial internal ApG-formation, at the subsequent step of incorporating the third nucleotide (UMP), the cRNA template would instead of translocating one position further forward, equilibrate to an energetically more favorable conformation by backtracking three positions (Figure 5). The primer formed by then would have re-aligned to a conformation similar to having initiated terminally but with one distinction: the 5΄-3΄ base pairing region would now favour translocating the template RNA when a trinucleotide primer has already been synthesized, be neutral at the stage of a tetra-nucleotide primer and would only start to hinder translocation with the incorporation of the fifth nucleotide. At this stage, when the 5΄-3΄ base-pairing would be similarly distorted as in terminal initiation, five new base-pairs (3AU, 2GC) could have been formed in contrast to only three at terminal initiation (Figure 5). Since we observe final full-length product RNA formation, this apparently compensates for breaking the strong 5΄-3΄ base pairing region thereby permitting elongation. However, it is possible that not only RNA-RNA interactions but also protein–RNA interactions are important in the choice of the position of initiation. This could be at the level of specific protein interactions with bases (e.g. at position 3 of the template, which is G in vPol and A in cPol) or in the role of the priming loop (8) which has been shown to be important in stabilizing the terminal initiation complex of vPol but is not for internal initiation by cPol (48).

Although internal initiation of cPol is slower than terminal initiation of RNA synthesis by vPol, its existence suggests it provides the virus with a certain advantage. *In vivo* it has been observed that ∼50% of virion RNA isolated from infected cells lacks one or two nucleotides at the 3΄ end (49). This demonstrates that the 3΄ extremity might be susceptible to nucleolytic cleavage or is incompletely synthesised. The latter might be the case since it is templated by the ultimate nucleotides of the 5΄ hook RNA. Thus while synthesizing the 3΄ end of the product RNA, fewer stabilizing interactions of the template RNA and polymerase are available upstream of the active site, perhaps resulting in premature termination of RNA synthesis. Since it less efficiently initiates RNA synthesis, any vRNA lacking nucleotides at the 3΄ end would be out-competed by complete vRNA to serve for the production of cRNA (Figure 6B). Hence, cRNA should predominantly consist of intact c5΄ ends but may lack nucleotides at the 3΄ end for the reasons just mentioned for vRNA. However, with the internal initiation mechanism accompanied by *prime-and-re-align*, the viral polymerase initiating at the cRNA promoter acquired a mechanism to repair minor deletions at the c3΄ end that would otherwise result in incomplete v5΄ ends. Apparently, the ability to produce intact v5΄ ends outweighs the associated reduction in rate.

Although our methodology allows detailed analysis of certain aspects of RNA synthesis, notably the differences between vPol and cPol, there are limitations to *in vitro* assays with a consequence that many important questions relevant to the RNP context of RNA synthesis or to the cellular context of the infected cell are not addressed. Our data support that by employing a primer complementary to the template RNA's ultimate and penultimate 3΄ end, an initiation complex is stabilized and the apparently rate-limiting formation of the first ApG-phosphodiester bond bypassed. Of note, full-length product RNA formation is recorded by our FP-based assay, the determined rate of which corresponds to the slowest, rate-limiting step of the entire RNA synthesis reaction. By bypassing the slowest rate-limiting step of ApG-formation, the subsequent rate-limiting step becomes apparent, which was demonstrated to correlate with the (strength of the) 5΄-3΄ base-pairing region in the promoter distal part. Since varying the strength of the 5΄-3΄ base-pairing region directly impacted the observed rate of RNA synthesis, we conclude that this step (breaking the 5΄-3΄ base-pairing region) is the next rate-limiting step of the complete reaction. We speculate that this step separates initiation from elongation. If elongation, or any step proceeding breaking the 5΄-3΄ base-pairing region would be slower, varying the 5΄-3΄ base-pairing region should not result in changes of the rate constants of full-length product RNA formation observed. Mechanistically, the only difference between primed and unprimed initiation of RNA synthesis is the accelerated frequency of the former reaction to proceed to final product-formation. With a catalytic rate about 300-fold improved for the primed over the unprimed initiation mechanism, our data argue that with a suitable capped RNA primer available, the influenza polymerase would almost exclusively perform transcription. So how does the polymerase ‘switch’ from transcription (capped RNA primed) to replication (unprimed) at the same vRNA promoter? A plausible explanation is based on the fact that influenza polymerase is known to be associated with host Pol II CTD (50,51), which likely gives it access to nascent transcripts for pirating (52). It is thus possible that Pol II CTD sites become saturated as infection proceeds, due to both increasing numbers of polymerase complexes and decreasing amounts of Pol II resulting from degradation (53) and, in the absence of available primers, RNPs replicate *de novo* instead. Thus the switch could be the result of the interplay of several factors at the systems level combined with intrinsic properties of the polymerase rather than the action of any single viral or host factor. Another interesting question relates to the relative amounts of cRNA and vRNA in infected cells. It is generally observed that cRNA (cRNPs) is in relatively low abundance (54,55) compared to vRNA (vRNPs). *In vitro*, we observe that vPol initiates replication considerably faster than cPol which would suggest that there should be an excess of cRNA over vRNA. This apparent contradiction could be explained by the fact that only a fraction of vRNPs are actually replicating (the others are transcribing or exported from the nucleus) or that viral and/or host factors might be recruited, accounting for elevated vRNP over cRNP levels (56).

In conclusion, the strengths of our newly developed, FP-based RNA synthesis assay are the defined conditions, reliability and effectiveness in high-throughput screening for inhibitors. As a proof of principle, we screened a set of compounds targeting RNA synthesis and found that cPol and vPol are differently inhibited by the 2΄F-2΄dNTP nucleoside analogues. This result could be exploited to develop novel influenza polymerase inhibitors (57).

## ACCESSION NUMBERS

Co-ordinates and structure factors for the influenza B polymerase co-crystallized with vRNA promoter and capped RNA primer are deposited in the wwwPDB with accession number 5MSG.

## Supplementary Material

Supplementary Data
