# Supplementary material for: An in vitro fluorescence based study of initiation of RNA synthesis by influenza B polymerase
Source: Nucleic Acids Res. 2017 Jan 25;45(6):3353–68. doi: 10.1093/nar/gkx043 (PMC5399792; doi:10.1093/nar/gkx043)
Supplement: Supplementary Data [file gkx043_supplementary_data.zip › nar-02699-m-2016-File012.pdf]

An *in vitro* fluorescence based study of initiation of RNA synthesis by influenza B polymerase

Stefan Reich[1,2], Delphine Guilligay[1,2,3] and Stephen Cusack[1,2,*]

[1] Grenoble Outstation, European Molecular Biology Laboratory, Grenoble, 38042, France
[2] Unit of Virus-Host Cell Interactions, EMBL-UGA-CNRS, Grenoble, 38042, France

* To whom correspondence should be addressed. Tel: 0033476207238; Email: cusack@embl.fr

Present Address:
[3] Delphine Guilligay, Institut de Biologie Structurale, CEA-UGA-CNRS, Grenoble, 38042, France

Supplementary Figures S1-S16

Supplementary Table S1

Supplementary References

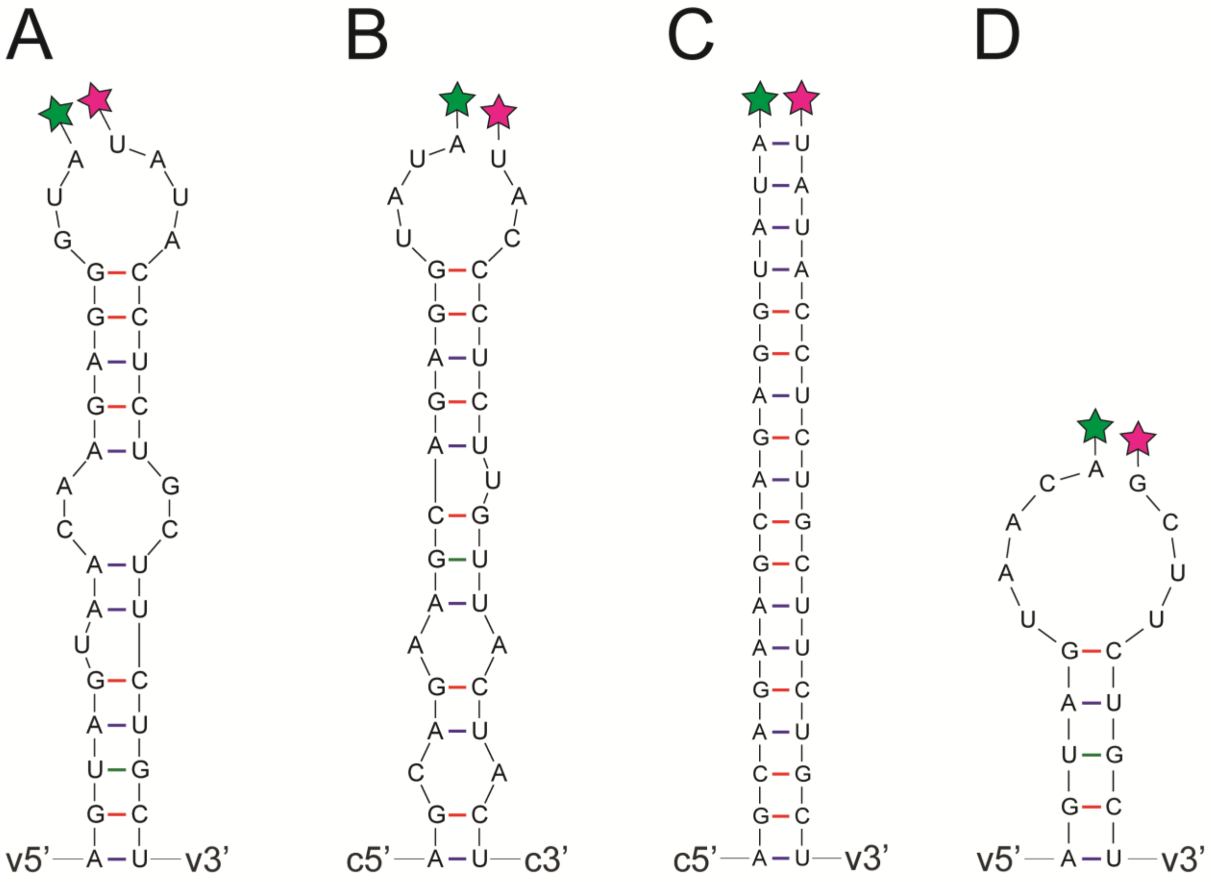

Figure S1: Predicted Influenza B virus promotor RNA secondary structures.

RNA secondary structures at 24 ºC were predicted using the DINAMelt (1,2) Web Server, specifically the “Two State melting” application (1). The 5′ end of the 5′ RNA as well as the 3′ end of the 3′ RNA are labeled and fluorophores at the distal ends schematically indicated by green and pink stars, representing FAM-Ex-5- and Cy3-fluorophores, respectively.

(A) Influenza B virus genomic promotor RNAs (vRNA) v5′ and v3′, each 18 nucleotides.

(B) Influenza B virus cRNA promoter (cRNA), comprising c5′ and c3′ RNAs (each 18 nucleotides).

(C) Influenza B virus hybrid promotor consisting of c5′ RNA and v3′ RNA, each 18 nucleotides.

(D) Influenza B virus genomic negative-strand promotor RNAs (vRNA) but with the v5′ RNA nucleotides 1-10 and the v3′ RNA nucleotides 1-9.

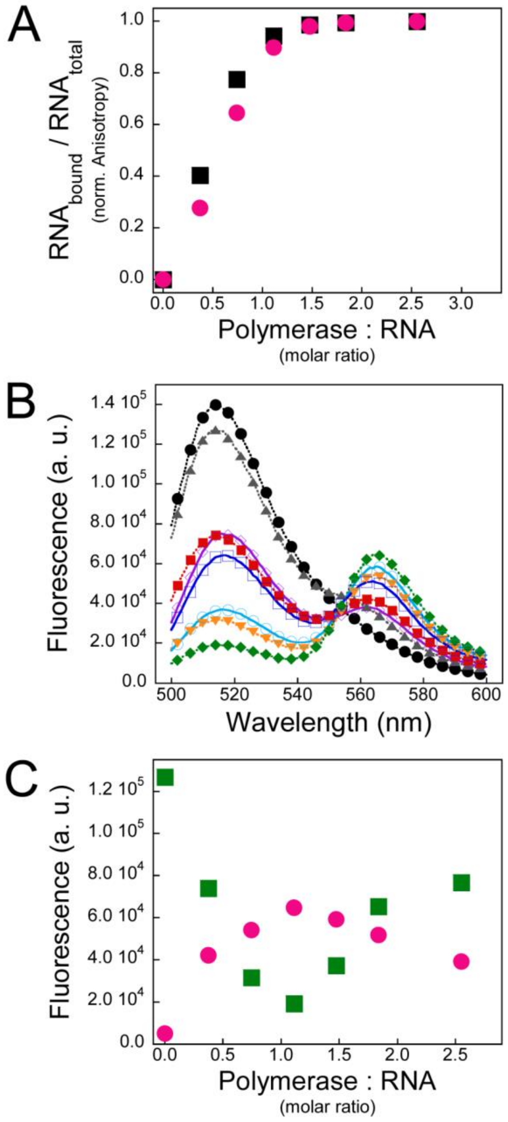

Figure S2: Both the 5′ and the 3′ promoter vRNA interact simultaneously with influenza B polymerase.

To monitor simultaneous binding of v5′ and v3′ to influenza B polymerase and obtain information on the stoichiometry, the predisposition of both RNAs to anneal and form a partially double-stranded RNA incapable of binding the polymerase (see Figure 1) required to be bypassed. This was achieved by applying the shorter v5′ nucleotides 1-10 which, compared to v5′ 1-18, showed strongly reduced affinity for the v3′ 1-18 (see Figure 1) and enabled to monitor both v5′ 1-10 and v3′ 1-18 interacting with polymerase. The v3′ 1-18 is Cy3-labelled at its 5′ end, the v5′ 1-10 is FAM-Ex-5-labelled at its 3′ end enabling detection of both RNAs separately. Concentration of

both RNAs was 0.5 µM, exceeding the previously determined K_D more than 10-fold to obtain information on the stoichiometry (see Figure 1).

(A) Utilizing the change in fluorescence anisotropy, both the v5′ 1-10 and the v3′ 1-18 interacted simultaneously with the influenza B polymerase and each in a one-to-one stoichiometry.

(B) Fluorescence emission spectra of 0.5 µM FAM-Ex-5-labelled v5′ 1-10 (black circles) and 0.5 µM Cy3-labelled v3′ 1-18 interacting with 0 µM (grey triangles), 0.19 µM (red squares), 0.37 µM (yellow triangles), 0.56 µM (green diamonds), 0.74 µM (light blue circles), 0.92 µM (blue squares) and 1.28 µM (purple diamonds) influenza B polymerase excited at 494 nm.

(C) Quantification of (B). The fluorescence intensities of the FAM-labelled v5′ 1-10 (Donor) and Cy3-labelled v3′ 1-18 (Acceptor) at the respective maxima on excitation at 494 nm plotted against the molar ratio of influenza B polymerase showed maximal FRET at a one-to-one-to-one molar ratio. Further increasing the polymerase-concentration resulted in decreased FRET, indicating heterogeneity with one or both RNAs bound to the same molecule of polymerase.

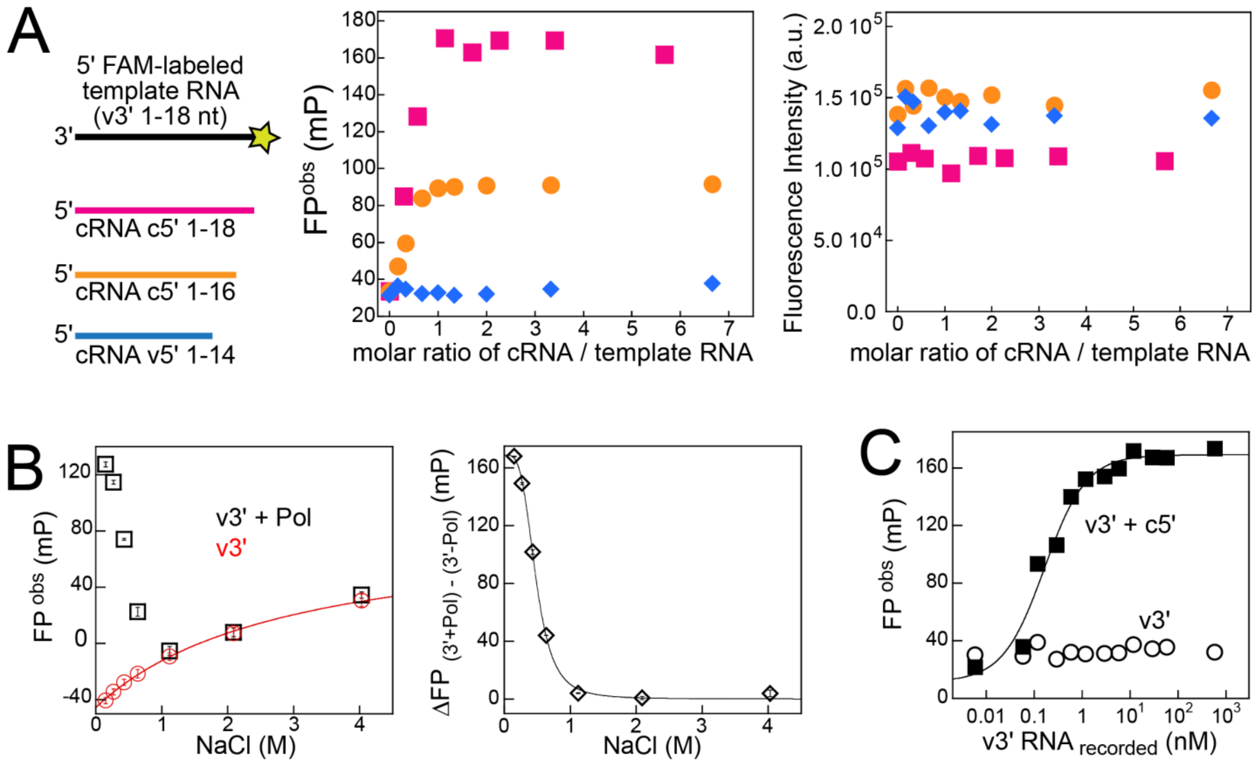

Figure S3: Details of the new FP-based RNA-synthesis assay.

(A) **Direct proportionality of the observed fluorescence polarization signal-changes to the complementary product / FAM-labelled template ratio and the sensitivity of the assay to the product RNA length**. FAM-Ex-5-labelled v3′ RNA nucleotides 1-18 (5′-FAM-Ex-5-UAUACCUCUGCUUCUGCU-3′; left panel, schematically and in black with the green star indicating the fluorophore) were incubated with perfect complementary c5′ RNA nucleotides 1-18 (5′-pAGCAGAAGCAGAGGUAUA-3′; pink), complementary c5′ RNA nucleotides 1-16 (5′-pAGCAGAAGCAGAGGUA-3′; yellow) or partially complementary v5′ RNA nucleotides 1-14 (5′-pAGUAGUAACAAGAG-3′; blue) in RNA synthesis assay buffer. As in the RNA synthesis assay, fluorescently-labelled template RNA concentration was constant 0.15 µM during the reaction and around 0.01 µM after quenching when recording the FP-signal. The observed FP-signal change was linearly dependent on the ratio of product-RNA added to fluorescently-labelled template RNA until reaching saturation at a one-to-one molar ratio. The full-length product RNA (c5′ 1-18) yielded a maximal signal-amplitude of ΔFP^{obs} ~ 130 while the shorter c5′ 1-16 yielded a reduced ΔFP^{obs} ~ 60 and v5′ 1-14 did not result in changes of the observed FP at the molar ratios indicated. Hence, the observed fluorescence polarization was most sensitive to full-length product RNA. Importantly, total fluorescence intensity ($I_{total} = I_{parallel} + 2*I_{perpendicular}$) remained constant (right panel).

(B) **Dissociation of influenza B polymerase and the fluorescently-labelled template v3′ RNA by increasing the ionic strength**. 0.15 µM FAM-Ex-5-labelled v3′ RNA nucleotides 1-18 (5′-FAM-Ex-5-UAUACCUCUGCUUCUGCU-3′) and 0.3 µM v5′ RNA nucleotides 1-14 (5′-pAGUAGUAACAAGAG-3′) were incubated with (black squares) or without (red circles) 0.25 µM influenza B polymerase in RNA-synthesis assay buffer at the indicated NaCl-concentrations for > 1 h at room temperature (T ~ 21 ºC). Samples were transferred to 384 well plates (FIA plate black, 128,0 / 85 mm, medium binding; Greiner Bio-One GmbH, Austria) and the FAM's fluorescence polarization recorded (excitation / emission = 485 / 520 nm with 10 nm band width; CLARIOstar, BMG Labtech, Germany), Black diamonds (right panel) correspond to the difference in fluorescence polarization of the v3′ in the presence and absence of influenza B polymerase at the indicated NaCl concentrations. Addition of 4M NaCl quenches the RNA-synthesis reaction and results in dissociation of template RNA from the polymerase. Mean values of duplicate experiments are plotted with the standard deviation indicated.

(C) **Detection limit of the FP-based RNA-synthesis assay**. 10 µM FAM-Ex-5-labelled v3′ RNA nucleotides 1-18 (5′-FAM-Ex-5-UAUACCUCUGCUUCUGCU-3′) with (black squares) and without (open circles) a twofold molar excess of c5′ RNA nucleotides 1-18 (5′-pAGCAGAAGCAGAGGUAUA-3′) were incubated in and serially diluted with RNA-synthesis assay buffer. 5 µl of each dilution were transferred to 80 µl 4.5 M NaCl before recording the fluorescence polarization signal (excitation / emission = 485 / 520 nm with 10 nm band width; CLARIOstar, BMG Labtech, Germany) in 384 well plates (FIA plate black, 128,0 / 85 mm, medium binding; Greiner Bio-One GmbH, Austria). For each concentration, the gain was adjusted to the sample with only the v3′ RNA. Plotted is the observed FP-signal against the final concentration of v3′ RNA recorded. Below a recorded concentration of ~ 1 nM, the FP-signal does not directly report on the fraction of c5′ product over v3′ template RNA anymore due to the equilibrium of single- and double-stranded RNA at these conditions (K_D ~ 0.03 nM).

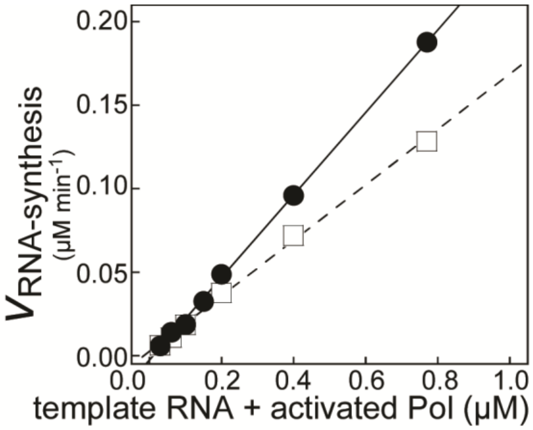

Figure S4: Linearity of the RNA synthesis assay.

Over a wide range, the rate of RNA synthesis was linearly proportional to the concentration of active enzyme, that is polymerase activated by v5′ RNA (nt 1-14) and bound to FAM-labelled v3′ template RNA (nt 1-18). The slope of the linear fit yielded k_{cat} ~ 0.25 min^{-1} at 0.5 mM NTPs (filled circles) and k_{cat} ~ 0.17 min^{-1} at 0.03 mM NTPs (open squares).

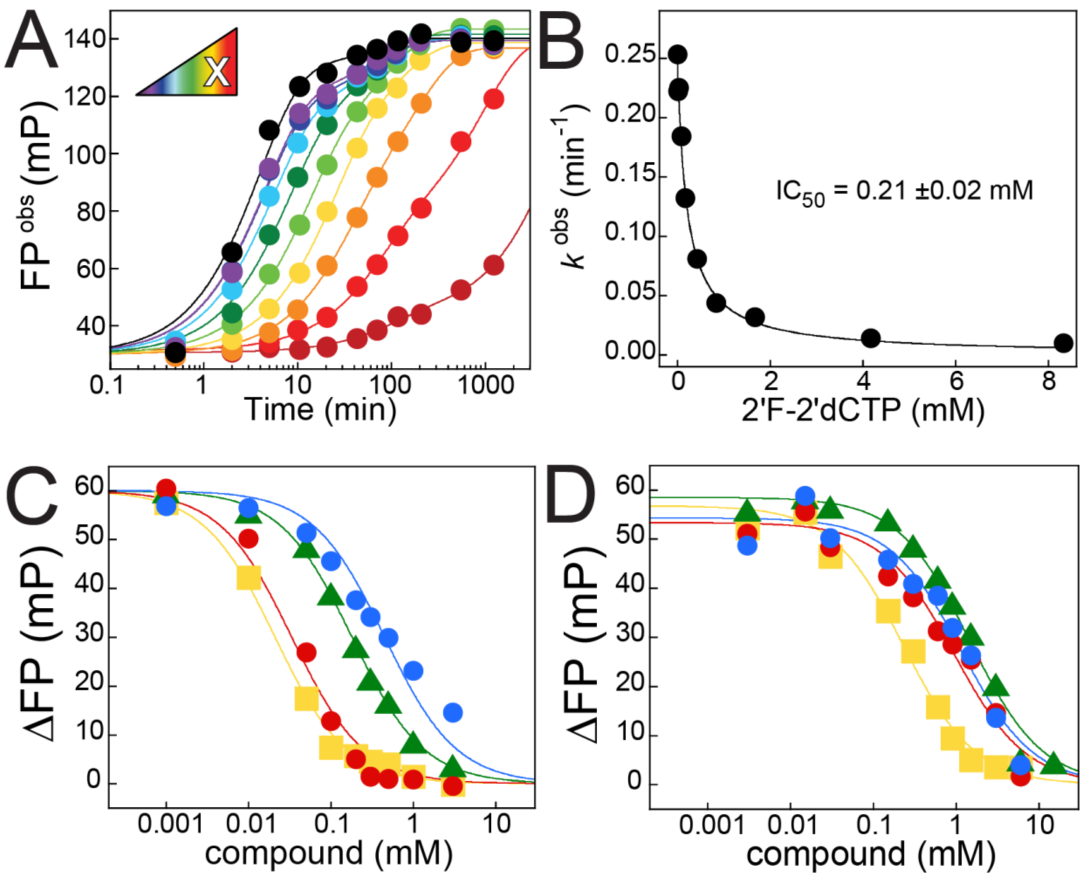

Figure S5: Inhibition of RNA synthesis by 2'F-2'dNTPs.

(A) **Quantification of the inhibiting effect of 2'F-2'dCTP (X) on influenza B polymerase catalyzed RNA synthesis reactions**. Shown are progress curves of constant 0.25 µM influenza B polymerase activated by an 1.2-fold molar excess of v5′ (nt 1-14), 0.15 µM labelled 3′ template RNA (v3′ nt 1-18), 0.5 µM capped RNA primer and 0.025 mM NTPs (each) in assay buffer supplemented with 1% (v/v) DMSO (black) at increasing concentrations of 2'F-2'dCTP (0.008 mM (purple), 0.025 mM (dark blue), 0.083 mM (light blue), 0.167 mM (dark green), 0.417 mM (light green), 0.83 mM (yellow), 1.67 mM (orange), 4.17 mM (red) and 8.33 mM (dark red)) at 24ºC. Time-dependent FP-signals (filled circles) were fitted double-exponentially according to pseudo-first order reactions since two phases were observed (straight lines).

(B) The observed rate constant of the dominant and fast phase (accounting for ~ 90% of the signal-amplitude) is plotted against the compound-concentration and hyperbolically decreases with increasing concentrations of 2'F-2'dCTP, yielding the IC_{50} = 0.21 ±0.02 mM.

(C) RNA synthesis was recorded for influenza B polymerase initiating at the vRNA promoter (v5′ nt 1-14, v3′ nt 1-18) and (D) at the cRNA promoter (c5′ nt 1-14, c3′ nt 1-18). Each reaction was performed with 0.25 µM polymerase activated by a 1.2 fold excess of 5′ RNA, 0.15 µM

fluorescently-labelled template RNA, 0.025 mM NTPs and saturating concentrations of capped RNA primer (0.5 µM and 2 µM for vPol and cPol, respectively) at T ~ 21 ºC in the presence of 2'F-2'dGTPs (yellow), 2'F-2'dCTPs (green), 2'F-2'dATPs (red) and 2'F-2'dUTPs (blue) at the concentrations indicated. RNA-synthesis catalyzed by vPol (C) and cPol (D) was quenched after 5 and 176 minutes, respectively. Fitting the data hyperbolically yielded the respective IC50-values (see Table 3).

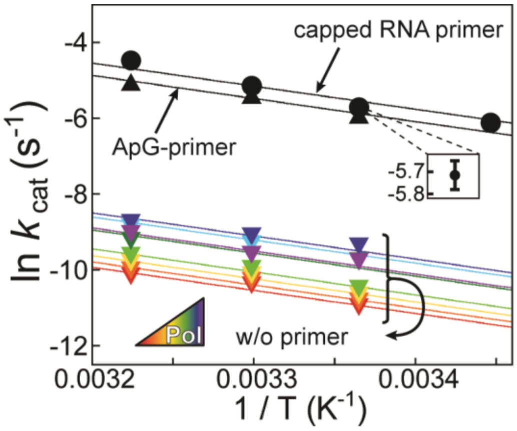

Figure S6: Dependence of the RNA synthesis on the reaction temperature.

Kinetics of RNA synthesis were recorded at the indicated temperatures and fitted according to pseudo-first order reactions, revealing the corresponding observed rate constant *k*. Analyzing the capped RNA-primed, the ApG-primed and the unprimed RNA synthesis reactions according to ARRHENIUS revealed all to require the same activation energy (E_A ~ 50 kJ mol^{-1}) and the reactions differing by their entropic contributions which can be interpreted as the frequency of successful collision of reactants. All reactions were performed at 0.15 µM fluorescently-labelled template RNA v3′ 1-18 and 0.5 mM NTPs (each). The capped RNA primed (1 µM) and ApG-primed (0.5 mM) reactions were catalyzed by 0.25 µM influenza B polymerase (activated by a 1.2-fold excess of v5′ 1-14). The unprimed RNA synthesis reactions were performed at 0.15 µM (red), 0.20 µM (orange), 0.30 µM (yellow), 0.50 µM (light green), 1.0 µM (dark green), 2.0 µM (light blue), 3.0 µM (dark blue) and 5 µM (purple) influenza B polymerase activated by a 1.2-fold excess of v5′ 1-14. Increasing the polymerase-concentration at “unprimed” reaction conditions is accompanied by an increase in the concentration of v5′ 1-14 that was demonstrated to serve as a primer (K_M ~ 1 µM, see Figure 3) and thus accelerated RNA synthesis. The inset shows the standard deviation from the mean of the capped RNA primed initiation of RNA synthesis of five independent experiments at 24ºC.

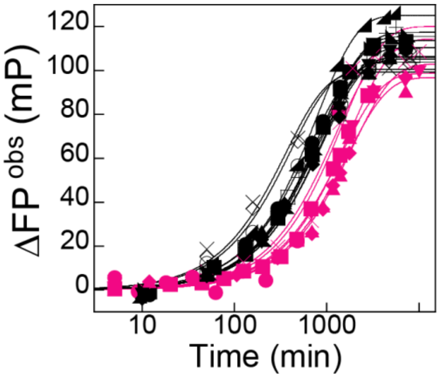

Figure S7: ***De novo* initiation of RNA-synthesis by influenza B polymerase from the vRNA and cRNA promoter.**

Progress curves of influenza B polymerase performing RNA-synthesis from the vRNA promoter (black symbols; vPol) and the cRNA promoter (pink symbols; cPol) in assay buffer at T = 24 ºC and 0.5 mM NTPs (each) at constant 0.15 µM FAM-Ex-5-labelled template RNA (nt 1-18) and 0.25 ±0.05 µM polymerase pre-incubated by a 1.2-fold molar excess of v5′ nt 1-14 or c5′ nt 1-14, respectively. For vPol and cPol, 12 and 6 progress curves are shown, respectively and fitted according to pseudo-first order reaction yielding the respective observed rate constants. The mean observed rate constants with the corresponding standard deviation are summarized in Table 1.

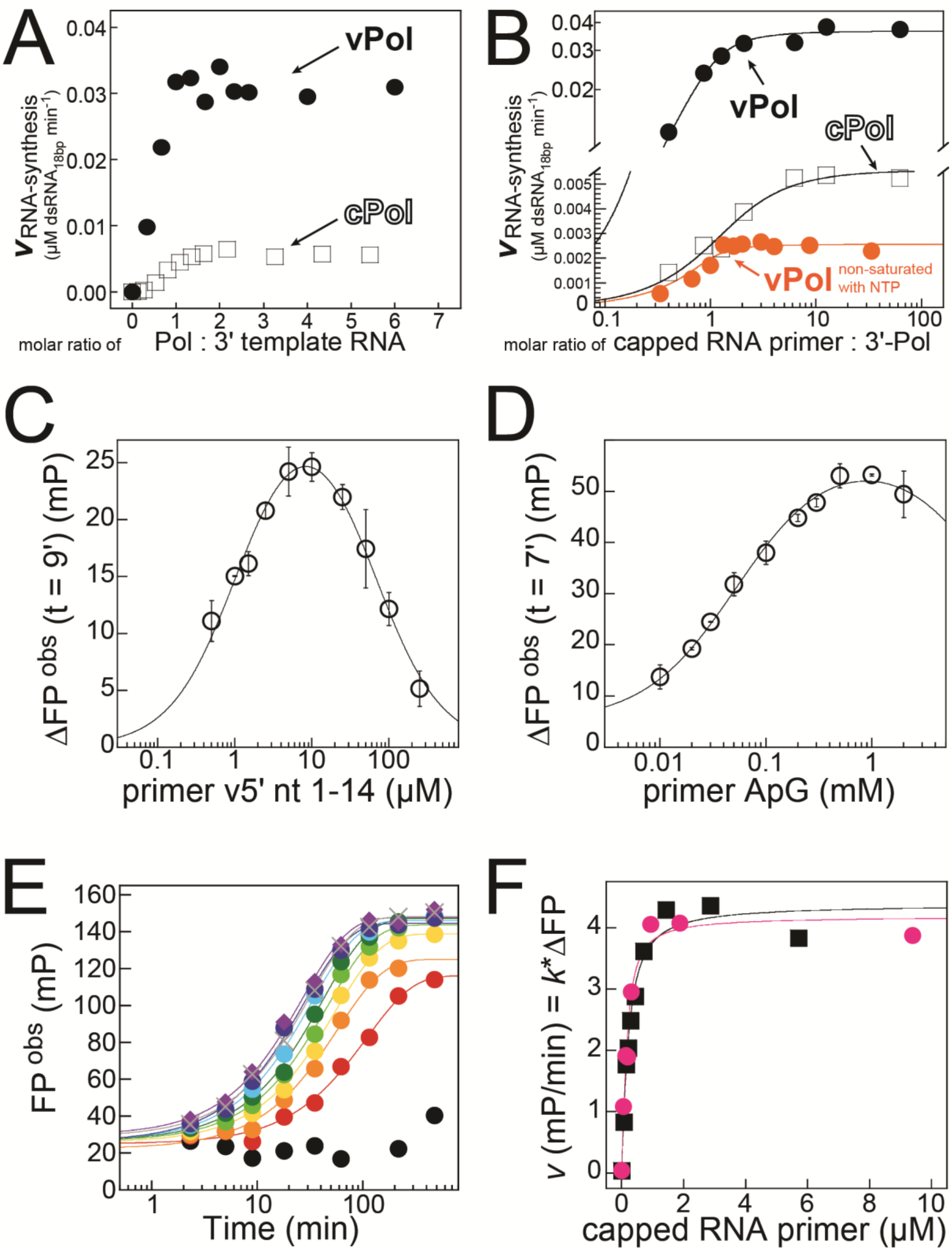

Figure S8: **Monomeric polymerase performs transcription-initiation and efficiently uses a capped RNA primer.**

(A) The rate of capped RNA primed initiation of RNA synthesis linearly increased for vPol (filled circles) and cPol (open squares) up to a one-to-one molar ratio of polymerase (activated by a 1.2-fold molar excess of v5′ 1-14 and c5′ 1-14, respectively) over the corresponding 3′ template RNA and remained constant thereafter. This indicated highly active protein preparation and an equimolar complex of polymerase and template RNA as the functional unit.

(B) At otherwise saturated reaction conditions, vPol (filled circles) reached maximum RNA synthesis-rates at a 1:1 stoichiometry of capped RNA primer over polymerase (activated by v5′ 1-14 and bound to v3′ template RNA) and revealed a high affinity for the primer at saturating 0.5 mM NTPs and at rate-limiting 0.0025 mM NTPs. The cPol (open squares) used the capped RNA primer less efficiently. Enzymatic parameters (K_M (capped RNA)) are summarized in Table 1.

(C) **K_M of RNA primer and vPol**. RNA-synthesis by influenza B polymerase initiating from the vRNA promoter (0.15 µM FAM-Ex-5-labelled v3′ RNA nt 1-18, 0.25 µM influenza B polymerase pre-incubated with a 1.2-fold molar excess of v5′ nt 1-14, 0.5 mM NTPs in RNA-synthesis assay buffer at T = 24 ºC) was recorded at the indicated concentrations of supplementary RNA primer v5′ nt 1-14. The FP-signal at a reaction time of 9 minutes after subtraction of the corresponding initial FP-signal (reactants mixed in the quenching solution) and fitted to a simple substrate-inhibition model (see Material and Methods). Shown are the mean values of duplicate experiments with the standard deviation indicated.

(D) **K_M of ApG and vPol**. RNA-synthesis by influenza B polymerase initiating from the vRNA promoter (0.15 µM FAM-Ex-5-labelled v3′ RNA nt 1-18, 0.25 µM influenza B polymerase pre-incubated with a 1.2-fold molar excess of v5′ nt 1-14, 0.5 mM NTPs in RNA-synthesis assay buffer at T = 24 ºC) was recorded at the indicated concentrations of ApG-primer. The FP-signal at a reaction time of 7 minutes after subtraction of the corresponding initial FP-signal (reactants mixed in the quenching solution) and fitted to a simple substrate-inhibition model with an offset of 5 mP (see Materials and Methods). Shown are the mean values of duplicate experiments with the standard deviation indicated.

(E) **Progress curves of RNA-synthesis by cPol at different concentrations of capped RNA primer**. RNA-synthesis was initiated by addition of 0.25 µM influenza B polymerase pre-incubated with a 1.2-fold molar excess of c5′ nt 1-14 to 0.15 µM FAM-Ex-5-labelled c3′ RNA nt 1-18, 0.5 mM NTPs and 0 (black), 0.07 (red), 0.14 (orange), 0.21 (yellow), 0.29 (light green), 0.43 (dark green), 0.71 (light blue), 1.43 (dark blue), 2.86 (purple diamonds) or 5.71 (grey crosses) µM capped RNA primer in RNA-synthesis assay buffer at T = 24 ºC and recorded and analyzed as described (see Material and Methods).

(F) **K_M of capped RNA primer and cPol**. Fitting the kinetics shown in E and of an analogue set of experiments (resulting in rates represented by black squares and pink circles, respectively) to pseudo-first order reactions yields observed rate constants and corresponding signal amplitudes, which in turn are proportional to the ratio of product RNA to template RNA. The initial rate (in mP/min) is plotted against the primer-concentration and fitted by the quadratic velocity equation (see Material and Methods) to obtain the K_M of capped RNA primer and cPol. With maximal FP-signal amplitude of ~ 110 mP (corresponding to ~ 0.15 µM product RNA), a maximal rate constant of ~ 0.04 min^{-1} is obtained.

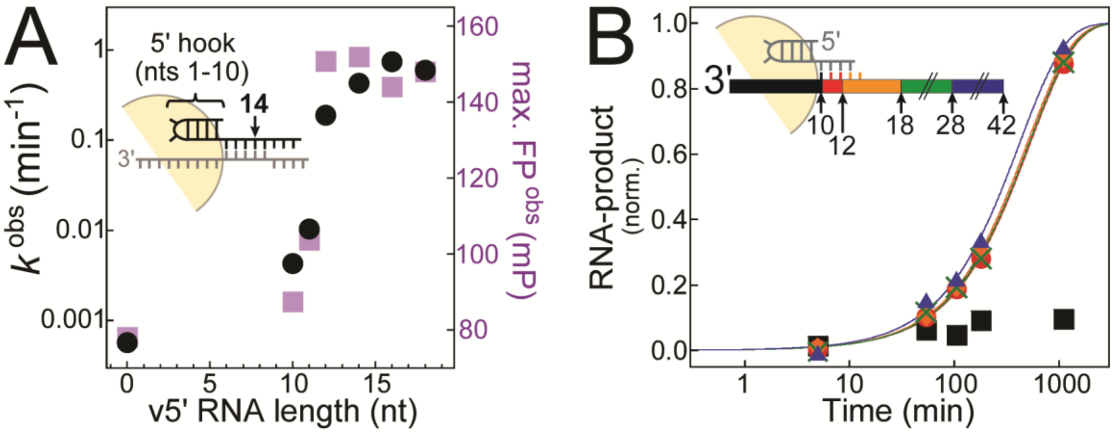

Figure S9. 5′ and 3′ vRNA requirements for optimal RNA synthesis activity.

(A) Nucleotides 1-10 of the very 5′ viral RNA end form an intra-molecular RNA-'hook'-conformation bound to a special pocket of influenza polymerase but only weakly activated the RNA synthesis capability. The v5′ nucleotides 1-14 were sufficient to completely activate the RNA-dependent RNA polymerization, both in terms of reaction rates (black circles) and product yield (purple squares). Fluorescently-labelled v3′ 1-18 was used as template RNA.

(B) Using v5′ 1-14 to activate influenza B polymerase, the requirements for template RNAs were investigated. The v3′ nucleotides 1-10 (black squares) were insufficient to serve as a template RNA but nucleotides 1-12 (red circles) were effectual and essentially required. Further increasing the length of template RNA at unprimed RNA synthesis conditions had no effect on the observed rate of full-length product-RNA synthesis (v3′ 1-18, orange diamonds; v3′ 1-28, green crosses; v3′ 1-42, blue triangles), highlighting the importance of the base-pairing between v5′ 11-13 and v3′ 10-12. For visualization purposes, the observed FP-signals were normalized since the different template RNAs yielded (slightly) different signal amplitudes.

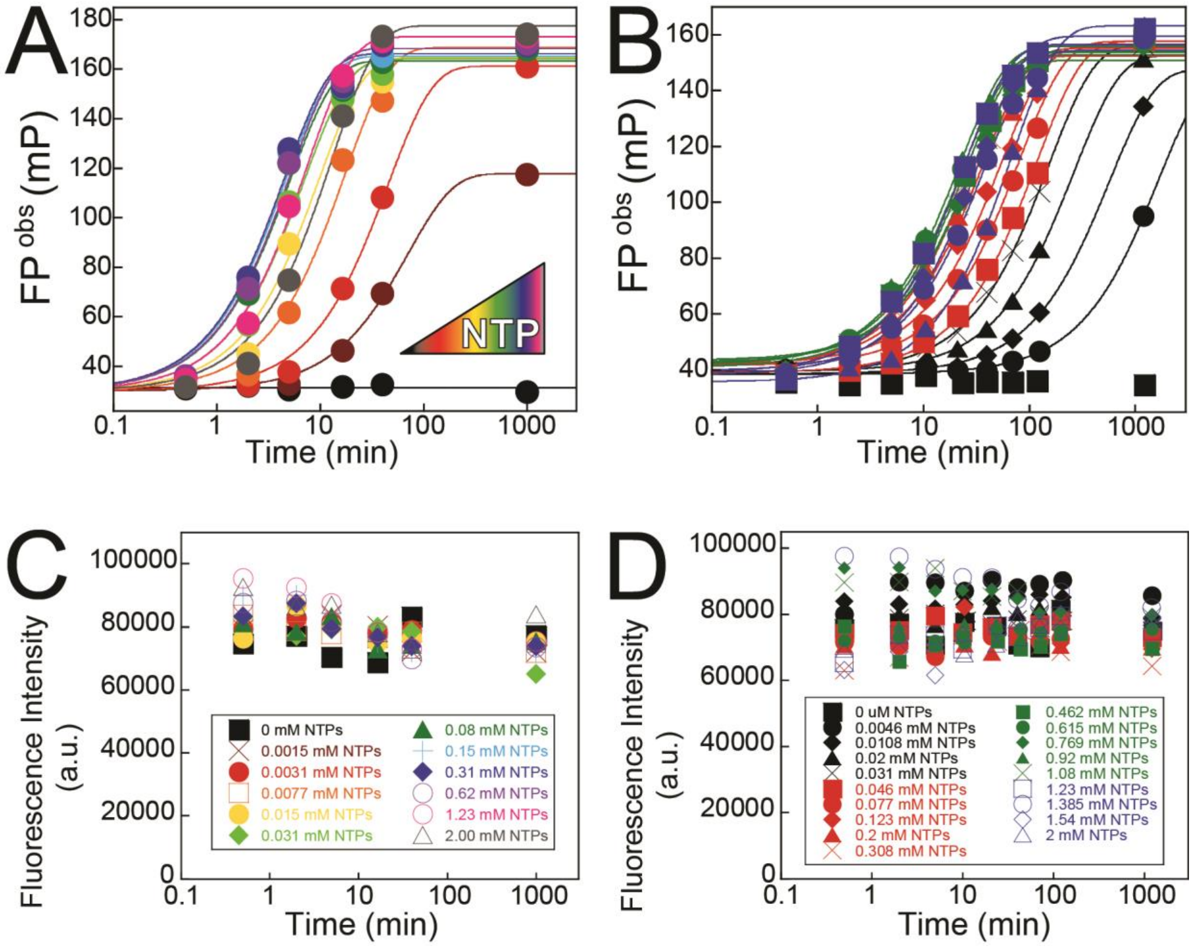

Figure S10: Progress curves of capped RNA primed initiation of RNA synthesis by influenza B polymerase from the vRNA and cRNA promoter at varying NTP-concentrations.

(A) Progress curves of 0.25 µM influenza B polymerase (incubated with a 1.2-fold molar excess of v5′ nucleotides 1-14; 5′-pAGUAGUAACAAGAG-3′), 0.15 µM 5′ FAM-labelled v3′ template RNA (5′-FAM-Ex-5-UAUACCUCUGCUUCUGCU-3′), 0.5 µM model capped RNA primer (5′-N7MeGpppAAUCUAUAAUAG-3′) and 0 mM (black), 0.0015 mM (brown), 0.003 mM (red), 0.008 mM (orange), 0.015 mM (yellow), 0.031 mM (light green), 0.077 mM (dark green), 0.154 mM (light blue), 0.308 mM (dark blue), 0.615 mM (purple), 1.23 mM (pink) and 2 mM (grey) NTPs each in assay buffer at T = 24 ºC. Kinetics were fitted according to pseudo-first order reactions and yielded the respective reaction rate constants. The derived observed rate constants are plotted against the respective NTP-concentration in Figure 6A (black upward triangles).

(B) Progress curves of capped RNA primed RNA synthesis of 0.25 µM influenza B polymerase activated by a 1.2-fold molar excess of c5′ RNA 1-14 and 0.15 µM fluorescently-labelled c3′ template RNA at 0 mM (black squares), 0.005 mM (black circles), 0.011 mM (black diamonds), 0.020 mM (black triangles), 0.031 mM (black cross), 0.046 mM (red squares), 0.077 mM (red circles), 0.123 mM (red diamonds), 0.200 mM (red triangles), 0.308 mM (red cross), 0.462 mM (green squares), 0.615 mM (green circles), 0.769 mM (green diamonds), 0.920 mM (green triangles), 1.08 mM (green cross), 1.23 mM (blue squares), 1.39 mM (blue circles), 1.54 mM (blue diamonds) and 2.00 mM (blue triangles) NTPs each at 2.1 µM model capped RNA primer in assay buffer at T = 24 ºC. Kinetics were fitted according to pseudo-first order reactions and yielded the respective reaction rate constants plotted in Figure 6A (pink downward triangles).

(C) and (D) Total fluorescence intensities for A) and B), respectively. The NTP-concentrations are indicated in the insert and the colouring correspond to A) and B), respectively.

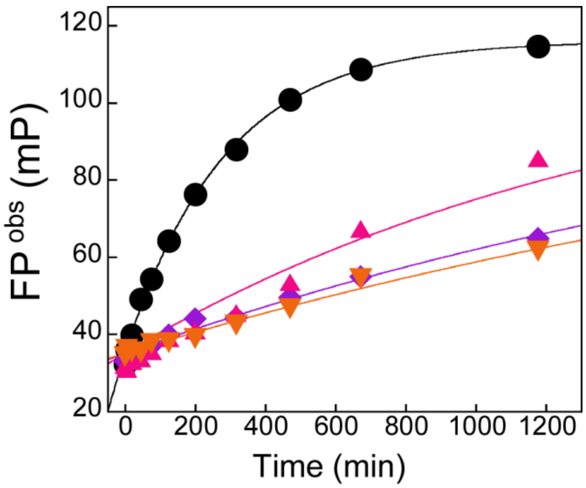

Figure S11: The effect of deleting the ultimate U1 from the vRNA and cRNA promoters' template RNAs on unprimed RNA synthesis.

Unprimed RNA synthesis catalyzed by 0.25 µM influenza B polymerase (activated by a 1.2-fold molar excess of v5′ 1-14 or c5′ 1-14, respectively) was recorded at 0.5 mM NTPs in assay buffer at T = 24 ºC and 0.15 µM of fluorescently-labelled template RNA v3′ 1-18 (black circles), v3′ 2-18 (purple diamonds), c3′ 1-18 (pink upward triangles) or c3′ 2-18 (yellow downward triangles). Deleting the ultimate U1 from the vRNA promoter's template RNA resulted in the decrease of the rate constant of RNA synthesis from $k_{v3'}$ = 0.0035 min^{-1} to $k_{v3'\text{-}U1}$ = 0.0004 min^{-1} while deleting the ultimate U1 from the cRNA promoter template RNA impaired the rate constant of RNA synthesis from $k_{c3'}$ = 0.00068 min^{-1} to $k_{c3'\text{-}U1}$ = 0.00035 min^{-1}.

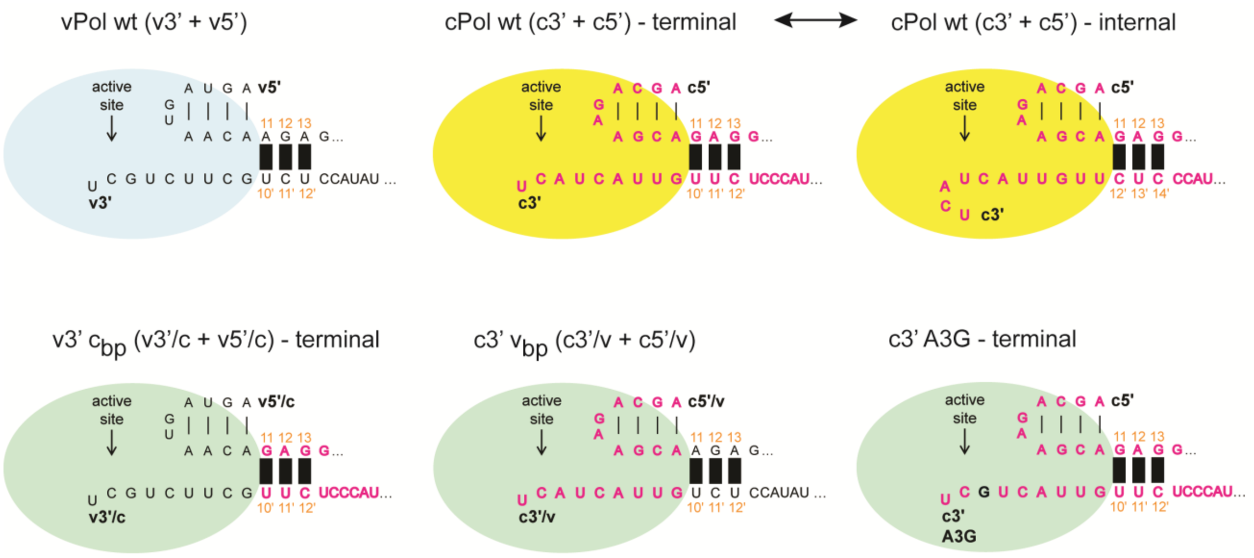

Figure S12: Schematic of influenza B polymerase bound to diverse sets of promoter RNAs.

Influenza B polymerase is depicted by the coloured ellipses and RNA-sequences of vRNA and cRNA origin in black and pink, respectively (see Supplementary Table 1). The 5′-3′ base-pairing region is indicated and nucleotides involved labelled. For polymerase bound to the cRNA promoter (yellow spheres), the equilibrium of the c3′ template RNA positioned for terminal or internal initiation of RNA synthesis is indicated.

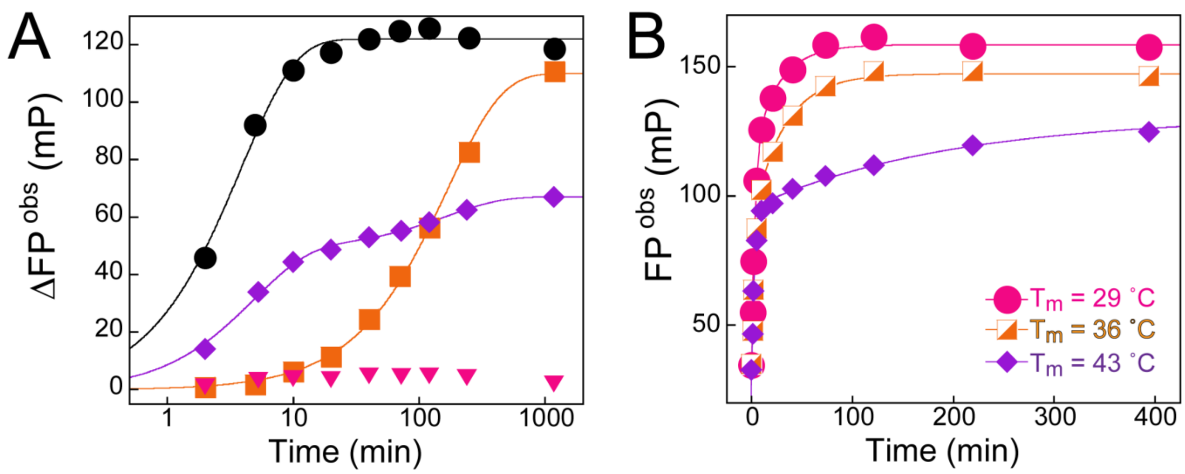

C

T_m (ºC)	v5'/v	c5'/c	v5'/c	c5'/v	v5'U6A/v	c5'A6U/c
v3'/v	18.5	70.5	39.8	54.2	31.4	63.8
c3'/c	57.5	23.6	50.8	30.4	46.9	38.9
v3'/c	32.1	60.3	27.4	67.1	46.7	52
c3'/v	43.3	39.5	61.8	12.5	30.2	52.1
c3'A3G/c	57.4	45.1	50.5	52.2	46.3	55.0
c3'A3G/v	42.6	57.6	62.0	35.8	28.7	65.9

Figure S13: Strengthened RNA-RNA interactions of variant promoter RNAs compete with RNA synthesis and may result in biphasic progress curves.

(A) Kinetics revealed fast RNA synthesis from the vRNA promoter (v3′ 1-18, v5′ 1-14; k_{vPol} = 0.26 min^{-1}; black circles) and slow RNA synthesis from the cRNA promoter (c3′ 1-18, c5′ 1-14; k_{cPol} = 0.006 min^{-1}; yellow squares) catalyzed by influenza B polymerase. The hybrid vRNA promoter consisting of v3′ 1-18 and c5′/v (c5′ nt 1-10 + v5′ nt 11-14; purple diamonds) showed biphasic kinetics with the fast and dominant phase proceeding with a vPol-like rate (k = 0.21 min^{-1}) but a reduced signal-amplitude (compared to vPol), indicating only a fraction of all template RNAs being transcribed. The hybrid cRNA promoter consisting of c3′ 1-18 and v5′/c (v5′ nt 1-10 + c5′ nt 11-14; pink triangles) did not yield detectable RNA synthesis. Both hybrid promoters are predicted to exhibit a similarly increased stability of intermolecular RNA-RNA interactions (T_m (v3′/v + c5′/v) = 54 ºC; T_m (c3′/c + v5′/c) = 51 ºC) since the 5′ hook RNA (nt 1-10) in each case is perfectly complementary to nucleotides 1-10 of the respective template

RNA. In all cases, RNA synthesis reactions were initiated by the addition of polymerase (activated by the 5′ RNA nt 1-14) to template RNA, primer and NTPs. In case of the hybrid vRNA promoter (but with the c5′ hook RNA), the fast vPol-like RNA synthesis proceeded until the v3′ and the c5′/v RNAs annealed which rendered the template RNA inaccessible for further RNA synthesis. In case of the hybrid cRNA promoter (but with the v5′ hook RNA), RNA synthesis apparently is slower than annealing of the c3′ and the v5′/c RNAs, thus disfavoring RNA synthesis in this kinetic partitioning. All reactions were carried out in 0.15 µM fluorescently-labelled template RNAs, 0.25 µM influenza B polymerase (activated by a 1.2-fold molar excess of the indicated 5′ RNA 1-14), 0.025 mM NTPs (each) and 2 µM capped RNA primer (saturating concentrations) in assay buffer at T = 24 ºC (see Materials and Methods).

(B) The effect of enhanced promoter RNA-RNA interactions on RNA synthesis was studied using in each case as template RNA fluorescently-labelled c3′A3G/v (5′-FAM-Ex-5-UAUACCUCUGUUACUGCU-3′) and influenza B polymerase activated by a 1.2-fold molar excess of v5′U6A/v (pAGUAGAAACAAGAG; pink circles), c5′/v (pAGCAGAAGCAAGAG; yellow squares) or v5′/v (pAGUAGUAACAAGAG; purple diamonds). All the variant promoters comprise of the vRNA promoter′s 5′-3′ base-pairing region and allow for fast terminal initiation of RNA synthesis but differ in the stability of their intermolecular RNA-RNA interactions with T_m (c3′A3G/v + v5′U6A/v) = 29 ºC; T_m (c3′A3G/v + c5′/v) = 36 ºC and T_m (c3′A3G/v + v5′/v) = 43 ºC (see panel C). With increasing (predicted) stability of the intermolecular RNA-RNA interactions, the biphasic nature of the progress curves evolved while the observed rate constant of the fast first phase remained constant at $k = 0.3 \pm 0.05$ min^{-1}, arguing for a kinetic partitioning of RNA synthesis and RNA-RNA-hybridization.

(C) RNA-RNA interactions were predicted using the “two-state melting (hybridization)” application of the DINAMelt Web Server (1,2) at T = 25 ºC and an RNA-concentration of 0.15 µM and are reported in terms of the respective melting temperature T_m (ºC). The respective 3′ template RNAs are indicated vertically, the 5′ RNAs horizontally with sequences summarized in Supplementary Table 1. Briefly, the slash separates 5′ RNAs in nucleotides 1-10 and 11-14 and 3′ RNAs in nucleotides 1-9 and 10-18 while v and c indicate the vRNA and cRNA origin, respectively.

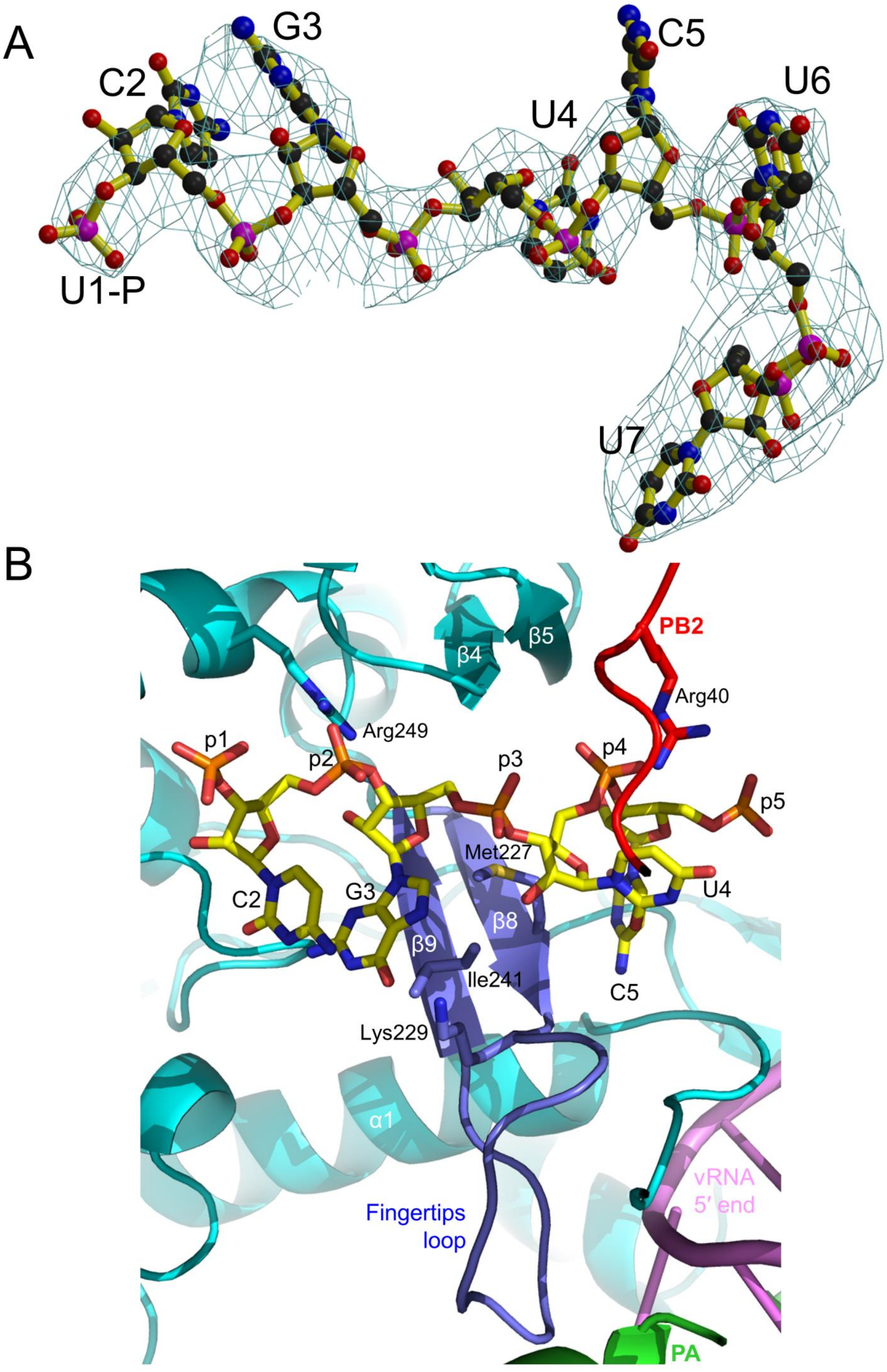

Figure S14: Conformation and interactions of the Influenza B vRNA 3′ end in the template entry tunnel.

(A) Omit Fo-Fc difference density for 3′ end nucleotides 1-7 (yellow sticks) contoured at 3.3 σ at 3.8 Å resolution.

(B) In the template entry tunnel 3′ end nucleotides 1-5 (yellow sticks) lie across the anti-parallel β-strands (β8 and β9) at the base of the fingertips loop (motif F, slate blue) of PB1 (cyan ribbons). PB1 Arg249 and PB2 Arg40 are positioned to interact with respectively the phosphates p2 and p4 of C2 and U4. Base G3 stacks on Ile241 and interacts with Lys229, while Met227 contacts the ribose of U4 (PB1 residues). All cited residues are absolutely conserved in all influenza polymerases.

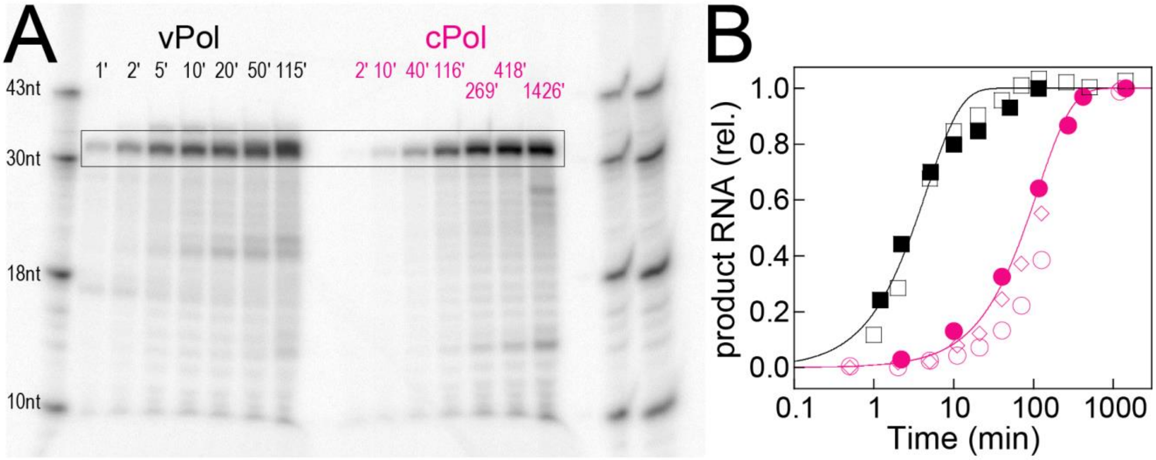

Figure S15: Comparison of RNA-synthesis followed by a conventional gel-based, radioactive assay and the new FP-based assay.

(A) RNA-synthesis was initiated by mixing 0.25 µM influenza B polymerase, pre-incubated by a 1.2 fold molar excess of v5′ nt 1-14 or c5′ nt 1-14, with 0.15 µM 5′-FAM-Ex-5-labelled template RNA v3′ nt 1-18 or c3′ nt 1-18, respectively, in assay buffer, saturating concentrations of the capped RNA primer ((N7MeGppp)AAUCUAUAAUAG-3′; 0.5 µM for vPol and 2 µM for cPol) and 0.02 mM NTPs (each) with traces of α^{32}P-labelled GTP (EasyTides, Perkin Elmer) at T = 24 ºC. At indicates times, aliquots of the reactions were quenched by and transferred to 2x RNA loading dye (8M Urea, 50 mM EDTA, bromphenol blue, xylene cyanol), incubated at T = 95 ºC for 5 minutes and separated by size in a denaturing polyacrylamide gel (8M Urea, 20% Acrylamide in TBE). Synthesis of radioactively labelled RNA was monitored via phosphorimaging (Typhoon). The dominant band corresponding to the full-length product RNA is highlighted by the box and was used for quantification of RNA-synthesis. An oligonucleotide ladder with sizes indicated was generated using T4 PNK (Fermentas), γ^{32}P-ATP and 43nt DNA, 30nt DNA, 18nt RNA and 10nt RNA according to standard protocols for 5′-end labelling.

(B) Semi-quantitative analysis of RNA-synthesis was performed with Fiji (ImageJ). Profile plots of the full-length product RNAs (as indicated by the box in (A)) of the raw image (8-bit greyscale; no background subtraction, no adjustments) were closed off vertically and the peak areas normalized to the respective maximum. RNA-synthesis by influenza B polymerase initiating at the vRNA promoter (vPol; black filled squares) or the cRNA promoter (cPol; pink closed circles) followed pseudo-first order rate laws and fitting yielded k_{vPol} =0.2 min^{-1} and k_{cPol} = 0.009 min^{-1}. Processing the raw image by subtracting background (e.g. sliding paraboloid, 100 pixel radius (apex)) before analysis yielded k_{vPol} =0.12 min^{-1} and k_{cPol} = 0.006 min^{-1} (not shown). Results

obtained by the traditional gel-based radioactive assay (filled symbols) are in agreement with the FP-based assay performed at identical reaction conditions (open squares and circles for vPol and cPol, respectively; open diamonds correspond to progress curves of cPol but at 0.03 mM NTPs).

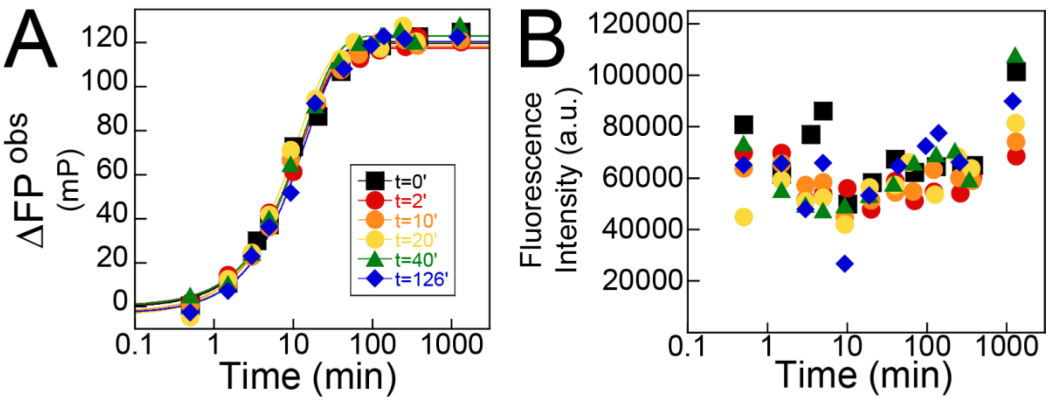

Figure S16: Pre-incubation of influenza B polymerase and cRNA promoter prior to initiation of RNA-synthesis by NTPs.

A) In assay buffer at T = 24 ºC, 0.25 µM influenza B polymerase was pre-incubated with 0.3 µM c5′ nt 1-14, 2 µM capped RNA primer and 0.15 µM 5′-(FAM-Ex-5)-labelled template RNA c3′ nt 1-18 (for sequences see Supplementary Table S1). After pre-incubation for 2 (red cicrles), 10 (orange circles), 20 (yellow circles), 40 (green triangles) and 126 (blue diamonds) minutes, the RNA-synthesis reaction was initiated by addition of final 0.5 mM NTPs (each). At a pre-incubation of 0 minutes (black squares), the reaction was initiated by addition of the template RNA. RNA-synthesis was recorded by the fluorescence polarization based assay as described in the Material and Methods. No impact of pre-incubating influenza B polymerase and the cRNA promoter on RNA-synthesis was observed.

B) Total fluorescence intensity signals of the reactions shown in A) (with identical coloring) remain constant while the reaction progresses. The scattering is attributed to different concentrations of fluorophore recorded.

Acronym	RNA-sequence
v5′/v	5′-pAGUAGUAACAAGAG-3′
c5′/c	5′-pAGCAGAAGCAGAGG-3′
v5′/c	5′-pAGUAGUAACAGAGG-3′
c5′/v	5′-pAGCAGAAGCAAGAG-3′
v5′U6A/v	5′-pAGUAGAAACAAGAG-3′
c5′A6U/c	5′-pAGCAGUAGCAGAGG-3′
v3′/v	5′-UAUACCUCUGCUUCUGCU-3′
c3′/c	5′-UACCCUCUUGUUACUACU-3′
v3′/c	5′-UACCCUCUUGCUUCUGCU-3′
c3′/v	5′-UAUACCUCUGUUACUACU-3′
c3′A3G/c	5′-UACCCUCUUGUUACUGCU-3′
c3′A3G/v	5′-UAUACCUCUGUUACUGCU-3′

Supplementary Table 1: Sequences of RNAs used.

RNA sequences used are listed with 5′ and 3′ ends indicated and "p" indicating mono-phosphate. To measure RNA synthesis with the described FP-based assay, 3′ template RNAs were modified at their 5′ end by FAM-Ex-5. All RNAs were purchased from IBA (Goettingen, Germany) in HPLC-grade purification. The acronyms v3′/v, c3′/c, v5′/v and c5′/c are identical to v3′ 1-18, c3′ 1-18, v5′ 1-14 and c5′ 1-14, respectively.

Supplementary References.

1. Markham, N.R. and Zuker, M. (2005) DINAMelt web server for nucleic acid melting prediction. *Nucleic acids research*, **33**, W577-581.
2. Markham, N.R. and Zuker, M. (2008) UNAFold: software for nucleic acid folding and hybridization. *Methods in molecular biology*, **453**, 3-31.
